# Water use strategies of *Populus euphratica* across age classes along groundwater depth gradients in the lower Tarim River

**DOI:** 10.3389/fpls.2026.1964884

**Published:** 2026-10-05

**Authors:** Guohua Li, Shuai Hao, Mao Ye, Xiaoqing Jiang, Zhongli Feng, Dingxue He

**Affiliations:** 1 College of Geography and Tourism, Xinjiang Normal University, Urumqi, China; 2 Xinjiang Laboratory of Lake Environment and Resources in Arid Zone, College of Geography and Tourism, Xinjiang Normal University, Urumqi, China

**Keywords:** ecological water conveyance, groundwater depth, intrinsic water-use efficiency, *Populus euphratica*, water source

## Abstract

How water source use and intrinsic water-use efficiency (iWUE) are linked across tree age classes under contrasting groundwater conditions remains unclear in desert riparian forests. We assessed water use strategies of young, middle-aged, and mature *Populus euphratica* in the lower Tarim River during ecological water conveyance (EWC) and non-EWC periods by combining hydrogen and oxygen stable isotopes of xylem and potential source waters with a Bayesian mixing model and leaf δ^13^C-derived iWUE. Across the five monitoring sections, mean groundwater depth was significantly lower during EWC than during non-EWC periods, averaging 4.7 and 4.9 m, respectively. At the Kunasite (DW) and Yingsu (F) sections, MixSIAR posterior mean contributions during EWC were 16.0–23.1% for groundwater, 14.4–17.6% for river water, and 6.6–19.0% for individual 40-cm soil layers. During non-EWC periods, posterior mean contributions from individual soil layers ranged from 10.9 to 20.3%, with relatively greater contributions from deeper layers. Associations between water source contributions and iWUE varied among age classes during EWC but were nonsignificant during non-EWC periods. Across sampling periods, mean iWUE was 58.29, 53.08, and 51.37 μmol mol^−1^ in young, middle-aged, and mature trees, respectively. The relationship between iWUE and groundwater depth also varied among sampling periods, with no consistent directional pattern. Based primarily on changes in the contributions of shallow and deep soil water along the groundwater depth gradient, we identified 4.5–6.0 m as a preliminary groundwater depth range associated with shifts in water source use by *P. euphratica*. These results show that water source use and iWUE in *P. euphratica* vary with hydrological conditions and tree age, providing empirical evidence for assessing its responses to EWC and groundwater variation in desert riparian forests.

## Highlights

- River water contributed in EWC, while deeper soil water contributed more in non-EWC.
- Young trees had the highest mean iWUE, with a larger sampling-period effect than age.
- Shallow and deep soil water responses indicated a preliminary GWD range of 4.5–6.0 m.

## 1 Introduction

Climate change is exacerbating drought and water scarcity, reshaping terrestrial ecohydrological processes in arid and semi-arid regions. How vegetation adjusts its water-use strategies in response to groundwater dynamics has therefore become a key question in ecohydrology (Rodriguez-Iturbe, 2000; Evaristo et al., 2015; Mu et al., 2021). Root water uptake and intrinsic water-use efficiency (iWUE) are sensitive indicators of plant responses to changing water availability (Li et al., 2025). Identifying the water sources used by dominant species, quantifying their iWUE, and clarifying the environmental controls on these processes are essential for understanding vegetation adaptation in arid regions and for guiding ecosystem restoration and water resource management.

The Tarim River Basin is a typical hyper-arid inland river basin, with annual precipitation below 50 mm and potential evaporation of 2000–3000 mm (Ding et al., 2024). In this chronically water-limited ecosystem, groundwater depth is a key hydrological factor shaping the distribution and succession of riparian vegetation (Chen et al., 2015; Wang et al., 2023). As a foundation tree species of desert riparian forests in the lower Tarim River, *P. euphratica* depends strongly on groundwater availability for growth and survival (Wan et al., 2022). Variation in groundwater depth can alter water acquisition and use by *P. euphratica*, with consequences for stomatal regulation, carbon assimilation, and iWUE (Ehleringer and Dawson, 1992; Zencich et al., 2002; Si et al., 2014). Moreover, *P. euphratica* trees of different age classes differ in root distribution, water-use patterns, and physiological regulation capacity (Liu et al., 2015; Jiang et al., 2025).

Stable isotopes of hydrogen, oxygen, and carbon provide time-integrated information on plant water acquisition, drought responses, and physiological performance (Snyder et al., 2022). Water isotopes, particularly δ^2^H and δ^18^O, are widely used to distinguish among soil water, groundwater, surface water, and xylem water, thereby supporting the identification of plant water sources (Dubbert and Werner, 2019). Compared with root excavation, sap flow measurements, and plant water potential measurements (De Deurwaerder et al., 2021; Schymanski and Or, 2017), isotope-based approaches are minimally destructive and can be combined with mixing models to estimate the proportional contributions of potential water sources (Brunel et al., 1995; Jiang et al., 2025). This approach assumes little or no fractionation of hydrogen and oxygen isotopes during root water uptake and xylem transport (Vega-Grau et al., 2021). Mixing models such as MixSIAR and IsoSource can then quantify the relative contributions of different water sources (Rothfuss and Javaux, 2017; Wang et al., 2019). From an ecological perspective, spatiotemporal variation in the use of water sources by plants reflects adjustments in water-acquisition strategies and may indicate hydrological niche differentiation along gradients from soil water to groundwater (Silvertown et al., 2015). The “Two Water Worlds” hypothesis proposes ecohydrological separation between water used by plants and mobile water contributing to groundwater recharge and streamflow (McDonnell, 2014).

Compared with instantaneous gas exchange measurements (Farquhar and Richards, 1984), leaf δ^13^C provides a time-integrated proxy for iWUE during leaf formation and is widely used to assess plant water-use responses to seasonal or long-term water limitation in arid regions (Farquhar et al., 1982; Ma et al., 2023). This proxy is based on the relationship between photosynthetic ^13^C discrimination in C_3_ plants and the ratio of intercellular to atmospheric CO_2_ concentration (*C_i_/C_a_*), which reflects the balance between carbon gain and transpirational water loss (Farquhar et al., 1982; Farquhar and Richards, 1984; Farquhar et al., 1989). However, iWUE estimates derived from leaf δ^13^C are sensitive to assumptions underlying carbon-isotope discrimination models (Petrík et al., 2023). In particular, the conventional *C_i_/C_a_* -based formulation does not explicitly account for finite mesophyll conductance (*g_m_*), while post-photosynthetic fractionation can introduce additional uncertainty when bulk leaf δ^13^C is used to estimate iWUE (Ma et al., 2021, 2023; Gimeno et al., 2021). Combined δ^13^C and δ^18^O measurements can further help distinguish the effects of photosynthetic carbon assimilation and stomatal regulation on iWUE variation (Chahem et al., 2025). Leaf oxygen isotope enrichment relative to xylem water (Δ^18^O=δ^18^O_leaf_−δ^18^O_xylem_) reflects evaporative enrichment of leaf water and is closely linked to stomatal regulation and transpiration (Barbour et al., 2004). However, studies on *P. euphratica* have largely examined water-source use, sap flow, and water-use efficiency separately, leaving unclear how belowground water acquisition is associated with leaf-level iWUE. This gap is particularly relevant in the groundwater-dependent riparian forests of the lower Tarim River, where EWC alters surface-water availability and groundwater conditions, while access to different water sources may vary with tree age. Consequently, age-related differences in water source partitioning and their relationship with iWUE along the groundwater-depth gradient remain poorly understood.

Accordingly, we hypothesized that (1) water source partitioning would respond to groundwater depth and EWC status differently among age classes, with greater river-water use during EWC and greater reliance on deeper water sources by mature than young trees; and (2) the relationship between water-source partitioning and iWUE would vary with tree age and EWC status. To test these hypotheses, we sampled young, middle-aged, and mature *P. euphratica* trees across five sections of the lower Tarim River. At two of these sections, δ^2^H and δ^18^O measurements of xylem water and potential source waters were used in the MixSIAR Bayesian mixing model to estimate source contributions. Leaf δ^13^C-derived iWUE was quantified across all sections. Groundwater depth was monitored across the study sections, while continuous sap-flow and meteorological observations at the DW section provided complementary information on plant water use and environmental conditions. We then examined water-source partitioning and iWUE along the groundwater-depth gradient to characterize their responses to groundwater variation and to identify a preliminary groundwater-depth range associated with water-source adjustment in *P. euphratica*.

## 2 Materials and methods

### 2.1 Study area

The lower Tarim River region is located in southeastern Xinjiang, China (39°26′23″–40°35′19″N, 87°33′42″–88°22′40″E; **Figure 1**), and represents a typical groundwater-dependent desert riparian ecosystem. Reduced upstream inflow and anthropogenic disturbance have caused long-term channel drying and groundwater decline. Since 2000, ecological water conveyance (EWC) has been implemented to restore riparian vegetation and maintain ecosystem function in the lower reaches of the Tarim River. From 2000 to 2025, 26 ecological water conveyance events were completed in the lower Tarim River, with a cumulative ecological water release of approximately 10.7 × 10^9^ m^3^ (Li and Li, 2026). Representative channel-water conditions during EWC and non-EWC periods are shown in **Supplementary Figure S1**. The vegetation is dominated by *P. euphratica*, with associated desert riparian species including *Tamarix ramosissima*, *Haloxylon ammodendron*, *Nitraria tangutorum*, *Glycyrrhiza uralensis*, and *Karelinia caspia*.

**Figure 1:**
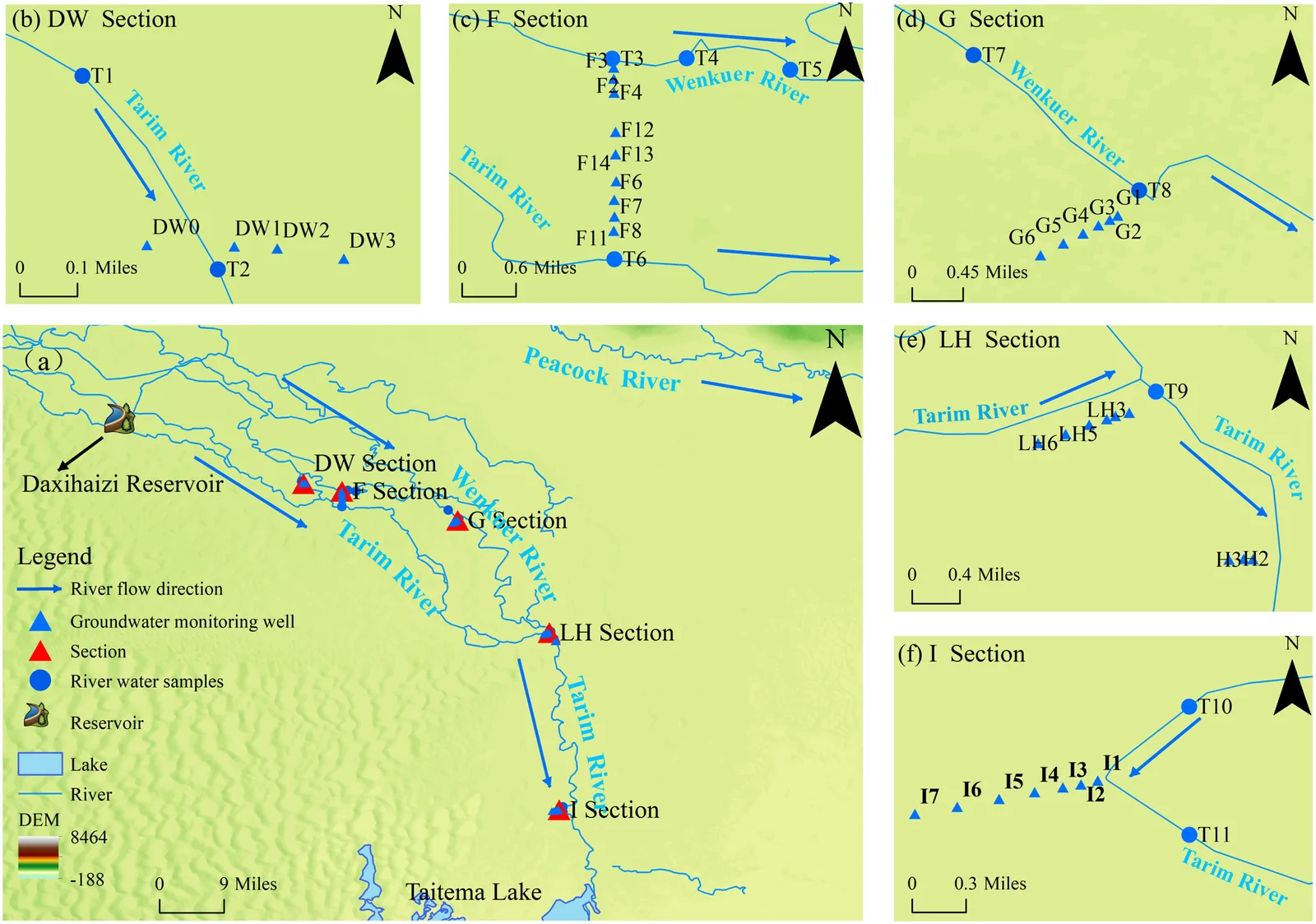
Sampling sites in the lower reaches of the Tarim River. The main map **(a)** shows the five sampling sections: Kunasite (DW), Yingsu (F), Kardeyi (G), Alagan (LH), and Yiganbujima (I). Insets **(b–e)** show the corresponding sampling layouts.

### 2.2 Sample collection

Field sampling was conducted around groundwater monitoring wells in the Kunasite (DW), Yingsu (F), Kardeyi (G), Alagan (LH), and Yiganbujima (I) sections. Samples were collected near each well in September 2023, July and October 2024, and May and August 2025. These campaigns covered the main growing season of *P. euphratica* over multiple years and captured both growing-season and interannual hydrological variation. Twig and leaf samples from *P. euphratica* trees of different age classes were collected for xylem water and leaf water extraction, respectively. Potential water sources, including soil water, groundwater, and river water, were sampled concurrently.

#### 2.2.1 Plant sample collection

Plant samples were collected between 08:00 and 10:00 local time (UTC + 8) on each sampling date. Trees representing three age classes were selected around groundwater monitoring wells across the five sections and at near-channel sites along the Tarim River. Trees were classified by DBH as young (DBH ≤ 10 cm), middle-aged (10 < DBH ≤ 20 cm), and mature (DBH > 20 cm), following thresholds previously applied to *P. euphratica* along the Tarim River (Keram et al., 2021). Current-year twigs and fully expanded, sun-exposed leaves were collected from healthy trees, immediately sealed, and stored in a cooler until laboratory processing. At each sampling site, one tree from each available age class was sampled for leaf δ^13^C analysis and iWUE estimation. Across the five sampling campaigns, 203 tree-level samples were collected, including 67 young, 71 middle-aged, and 65 mature trees (**Supplementary Table S1**).

#### 2.2.2 Sampling design for water-source partitioning

Six 20 m × 20 m plots were established at the DW and F sections for stable isotope-based water source partitioning. At DW, the plots were located 0–5, 200–205, and 400–405 m from the river channel, whereas those at F were located 0–5, 800–805, and 2995–3000 m from the channel. Within each plot, three *P. euphratica* trees were sampled for each age class present, giving a total of 48 trees (12 young, 18 middle-aged, and 18 mature; **Supplementary Table S2**). For each age class represented within a plot, soil was sampled from one profile adjacent to the selected trees to a depth of 2 m. Samples were collected from five 40-cm depth intervals, immediately sealed, and stored at low temperature until water extraction to minimize evaporation-induced isotopic alteration. Across the two sampling campaigns, 160 soil samples were collected.

#### 2.2.3 Groundwater and river water sample collection

Groundwater and river water samples were collected with a stainless-steel sampler. Groundwater was sampled from each monitoring well (**Supplementary Table S3**), whereas river water was collected from the natural river channel at approximately 20 cm below the water surface. Before sampling, the sampler and bottles were rinsed with the water to be collected; the bottles were then filled, sealed with Parafilm, and stored at low temperature. Total dissolved solids (TDS), electrical conductivity (EC), pH, dissolved oxygen (DO), water temperature, and salinity were measured *in situ* using a portable multiparameter water quality meter (HQ4300, Hach, USA).

#### 2.2.4 Fixed *in situ* monitoring system

A continuous field monitoring system was established at the DW section in May 2025 to characterize short-term plant–environment interactions. An automatic weather station recorded air temperature, relative humidity, wind speed, and solar radiation, with their temporal variations shown in **Supplementary Figure S2**. Groundwater depth was monitored hourly using level loggers (HOBO U20-001-01, Onset Computer Corporation, USA) installed in wells located 50, 100, 200, and 250 m from the river channel. Sap flow was recorded at 15-min intervals using thermal dissipation systems (FLGS-TDP, Dynamax, USA) installed on *P. euphratica* trees of different age classes approximately 20 m from the riverbank.

### 2.3 Sample processing

#### 2.3.1 Determination of basic physicochemical properties of soil and leaves

All laboratory analyses were performed at the Laboratory of Lake Environment and Resources in Arid Regions, Xinjiang. Soil water content (SWC) was determined gravimetrically. After plant residues and gravel were removed, 20.0 g of fresh soil was weighed and oven-dried at 105 °C to constant weight. For soil salinity, electrical conductivity, and pH measurements, 20.0 g of air-dried soil was mixed with 100 mL of deionized water, shaken for 30 min, allowed to stand for 30 min, and filtered. Leaf water content (LWC) was also determined gravimetrically: 10.0 g of fresh leaves was weighed and oven-dried at 70 °C to constant weight before LWC was calculated using Equations 1 and 2, respectively.

(1) $$\text{SWC}(\%)=\frac{\text{FW}-\text{DW}}{\text{DW}}\times 100$$

(2) $$\text{LWC}(\%)=\frac{\text{FW}-\text{DW}}{\text{DW}}\times 100$$

where FW is fresh weight (g) and DW is dry weight (g).

#### 2.3.2 Hydrogen and oxygen stable isotope analysis of water samples

Water was extracted from plant and soil samples using an automated cryogenic vacuum extraction system (Li-2200, LICA United Technology, Beijing, China). The extracted water and field-collected water samples were filtered through 0.45-μm cellulose acetate membranes and analyzed for δ^2^H and δ^18^O using a liquid water isotope analyzer (DLT-100, Los Gatos Research, USA). Each sample was injected six times; the first two injections were discarded to minimize memory effects, and the mean of the final four injections was used for analysis. Plant water isotope data were corrected for organic contamination following Gai et al. (2023). Isotope values were reported in per mil (‰) relative to Vienna Standard Mean Ocean Water (VSMOW) and calculated using Equation 3:

(3) $$\delta (‰)=({\text{R}}_{\text{sample}}/{\text{R}}_{\text{standard}}-1)\times 1000$$

where δ is the isotopic composition, and R_sample_ and R_standard_ are the *^2^H/^1^H* or *^18^O/^16^O* ratios of the sample and standard, respectively. Analytical precision was ±0.5‰ for δ^2^H and ±0.15‰ for δ^18^O.

#### 2.3.3 Determination of leaf δ^13^C and total carbon and nitrogen contents

Leaf samples of *P. euphratica* were rinsed with ultrapure water, air-dried, and then oven-dried at 70 °C to constant weight. The dried samples were ground, passed through a 100-mesh sieve, and homogenized for subsequent analysis. Total carbon (C) and nitrogen (N) contents were determined using an elemental analyzer (Flash EA1112, Thermo Fisher Scientific, USA). Stable carbon isotope analysis was performed at the Key Open Laboratory of Tree-ring Physical and Chemical Research, China Meteorological Administration. Approximately 0.4–0.6 mg of homogenized leaf powder was weighed, sealed in tin capsules, and combusted in an elemental analyzer to produce CO_2_. After purification, the CO_2_ was introduced into an isotope ratio mass spectrometer (Delta V Advantage, Thermo Fisher Scientific, USA) to determine the carbon isotope composition. Analytical precision was within ±0.2‰. The results were reported relative to the Vienna Pee Dee Belemnite (VPDB) standard, and δ¹³C values were calculated using Equation 4:

(4) $$\delta^{13}\text{C}(‰)=({\text{R}}_{\text{sample}}/{\text{R}}_{\text{standard}}-1)\times 1000$$

where δ^13^C is the carbon isotope composition of the sample relative to the VPDB standard, and R_sample_ and R_standard_ are the *^13^C/^12^C* ratios of the sample and standard, respectively.

### 2.4 Analytical methods

#### 2.4.1 iWUE calculation for *P. euphratica*

Leaf carbon isotope discrimination (Δ^13^C) was calculated from the δ^13^C values of atmospheric CO_2_ and leaf samples following Farquhar and Richards (1984) (Equation 5):

(5) $$\delta^{13}\text{C}=\frac{{\text{δ}}^{13}{\text{C}}_{\text{air}}-{\text{δ}}^{13}{\text{C}}_{\text{leaf}}}{1+{\text{δ}}^{13}{\text{C}}_{\text{leaf}}/1000}$$

where δ^13^C_air_ and δ^13^C_leaf_ are the carbon isotope compositions of atmospheric CO_2_ and leaf samples, respectively. Following previous studies (Farquhar et al., 1989; Sparks and Ehleringer, 1997; Ma et al., 2023), δ^13^C_air_ was set to −8‰.

The ratio of intercellular to atmospheric CO_2_ concentration (*C_i_/C_a_*) was derived from Δ^13^C (Equation 6):

(6) $$\frac{{\text{C}}_{\text{i}}}{{\text{C}}_{\text{a}}}=\frac{\delta^{13}\text{C}-\text{a}}{\text{b}-\text{a}}$$

where a and b are the isotope fractionation factors associated with CO_2_ diffusion through stomata and Rubisco carboxylation, respectively. Values of 4.4‰ and 27‰ were used for a and b, respectively (Farquhar and Richards, 1984; Condon et al., 2004; Jia et al., 2022).

iWUE was then calculated using Equation 7:

(7) $$\text{iWUE}=\frac{{\text{C}}_{\text{a}}(1-{\text{C}}_{\text{i}}/{\text{C}}_{\text{a}})}{1.6}$$

where *C_a_* denotes atmospheric CO_2_ concentration. Monthly mean *C_a_* values for each sampling campaign were obtained from the global monthly mean atmospheric CO_2_ record of the NOAA Global Monitoring Laboratory (Lee et al., 2018; Wu et al., 2024), with the values used in the calculations provided in **Supplementary Table S4**. Atmospheric *δ^13^C_a_* was fixed at −8‰ for the primary calculations. To assess the sensitivity of iWUE estimates to this assumption, the calculations for September 2023, July 2024, and October 2024 were repeated using the corresponding monthly background *δ^13^C_a_* values from the Scripps CO_2_ Program (**Supplementary Table S5**). Because mesophyll conductance (*g_m_*) was not measured, iWUE was estimated using the conventional Farquhar model based on *C_i_/C_a_* and is reported as leaf δ^13^C-derived iWUE.

General linear models were used to examine variation in leaf δ^13^C-derived iWUE. Age class and sampling period were included as categorical fixed factors. For samples with corresponding groundwater depth measurements, GWD was mean-centered and included as a continuous covariate. The age class × sampling period interaction and the interactions of GWD with age class and sampling period were tested by comparing nested models with F-tests. Samples from near-channel sites were excluded from models containing GWD because groundwater depth measurements were unavailable for these sites. Partial η^2^ was calculated for the main effects of age class and sampling period. Model assumptions were assessed visually using plots of residuals versus fitted values and normal Q–Q plots (**Supplementary Figure S3**). All models were fitted in Python using statsmodels version 0.14.6.

#### 2.4.2 Analysis of water sources used by *P. euphratica*

The proportional contributions of potential water sources to *P. euphratica* xylem water were estimated using the Bayesian isotope mixing model MixSIAR (version 3.1.12) in R. River water, groundwater, and soil water from different depths were specified as sources, and xylem water was used as the mixture. Source isotope data were entered as mean δ^2^H and δ^18^O values with corresponding SDs (**Supplementary Table S6**). Isotopic discrimination factors for both δ^2^H and δ^18^O were set to 0‰, assuming negligible isotopic fractionation between source and xylem water. The default uninformative Dirichlet prior (α = 1 for each source) was used for source proportions. The model was run using the “extreme” MCMC setting and the “Process only” error structure. Model convergence was assessed using the Gelman–Rubin and Geweke diagnostics (Stock et al., 2018; Kim et al., 2023).

## 3 Results and analysis

### 3.1 Variation in groundwater depth, plant water content, and soil water content in the lower Tarim River

EWC generally raised the groundwater table and increased SWC in *P. euphratica* habitats (**Figure 2**).

**Figure 2:**
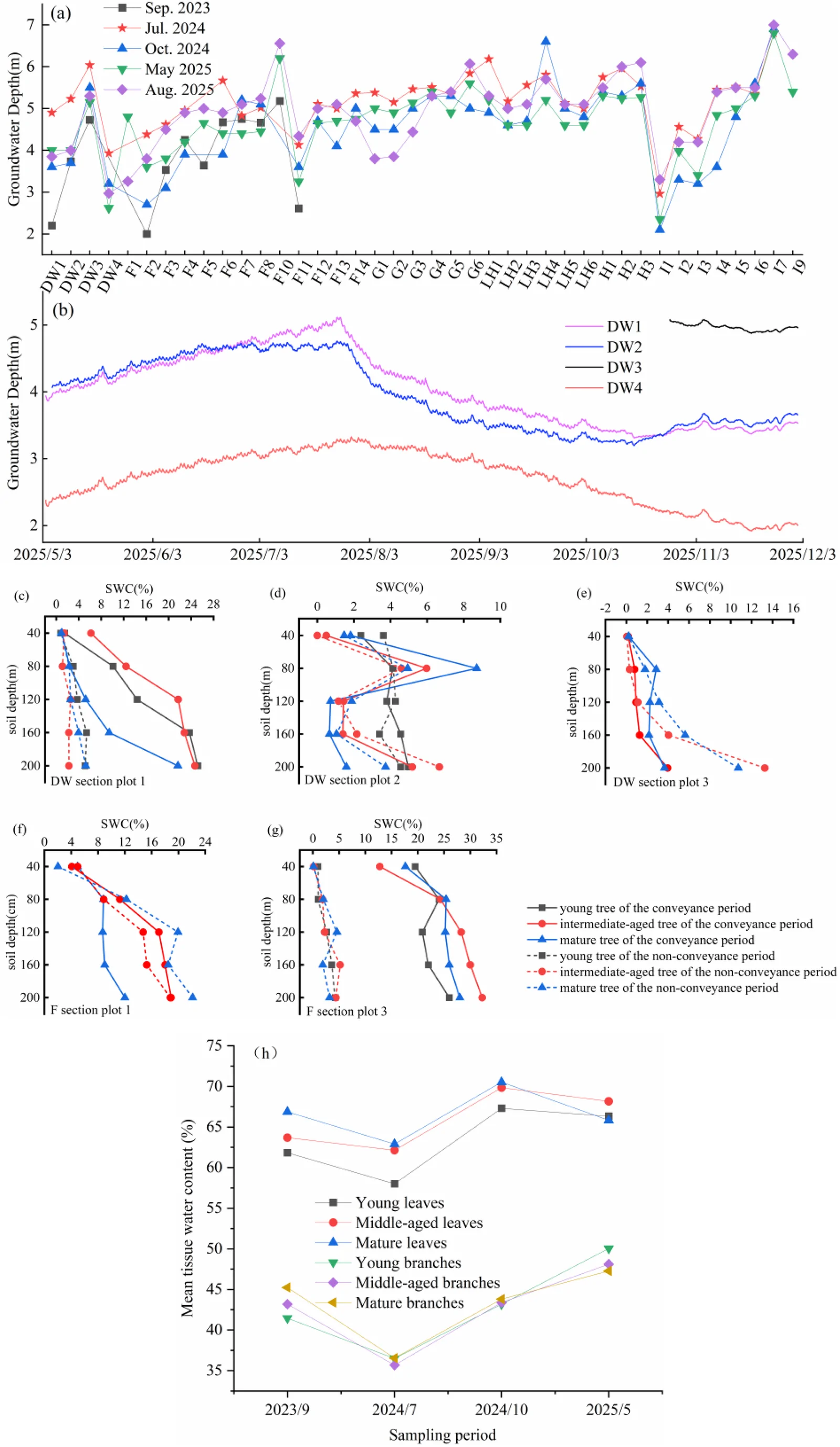
Spatiotemporal variation in groundwater depth **(a, b)**, SWC **(c–g)**, and branch and leaf water content of *P. euphratica* **(h)** in the lower Tarim River. **(a)** Groundwater depth at monitoring wells across the five sections during the five sampling campaigns; **(b)** hourly groundwater depth at the DW section during May–October 2025; **(c–e)** SWC profiles near *P. euphratica* trees of different age classes in Plots 1–3 at DW; and **(f, g)** SWC profiles in Plots 1 and 3 at F.

Groundwater depth differed across sections and sampling periods along the lower Tarim River (**Figure 2a**). Across the 39 monitoring points, mean groundwater depth was 4.65 ± 0.96 m during EWC and 4.92 ± 0.76 m during non-EWC periods. The difference between the two hydrological periods was significant (paired *t*-test, *t*_38_ = 3.50, *P* = 0.001), with a mean paired difference of 0.27 m. The magnitude of this difference varied among sections. Continuous monitoring at DW further showed that mean groundwater depth decreased from 4.54 to 3.74 m at DW1, from 4.51 to 3.53 m at DW2, and from 2.90 to 2.76 m at DW4 between May–July and August–October 2025 (**Figure 2b**).

SWC showed clear vertical variation at the DW and F sections and differed between the EWC and non-EWC periods (**Figures 2c–g**). Overall, SWC was higher during the EWC period, although the magnitude of the response varied among sections, plots, and tree age classes. At DW, SWC increased mainly in the middle and deep soil layers, particularly at 80–200 cm in Plot 1, whereas Plot 2 had lower SWC and greater vertical variability. At F, the strongest increase occurred in Plot 3, especially at 80–200 cm, likely because this plot was closer to the Qiwenkuoer River and occupied a lower topographic position, making the soil profile more susceptible to river-water recharge.

Mean leaf water content was consistently higher than branch water content across the sampling periods examined (**Figure 2h**). When age classes were pooled within each sampling period, mean leaf water content ranged from 61.39 ± 5.02% in July 2024 to 69.36 ± 4.89% in October 2024, whereas branch water content ranged from 36.20 ± 2.68% in July 2024 to 48.16 ± 7.73% in May 2025. Paired comparisons showed higher leaf than branch water content in September 2023 (*t*_15_ = 16.30), July 2024 (*t*_15_ = 18.65), and October 2024 (*t*_39_ = 29.17; all *P* < 0.001), with the same difference detected in May 2025 (Wilcoxon signed-rank test, *W* = 0, *P* < 0.001). Among age classes, mean leaf water content was highest in mature trees in September 2023, July 2024, and October 2024, and in middle-aged trees in May 2025.

### 3.2 Seasonal and age-related variation in water sources used by *P. euphratica*

Posterior mean estimates of water source contributions showed numerical variation between EWC and non-EWC periods, among plots, and among tree age classes at the DW and F sections (**Figure 3**). The corresponding 95% credible intervals (CrIs) are provided in **Supplementary Table S7**.

**Figure 3:**
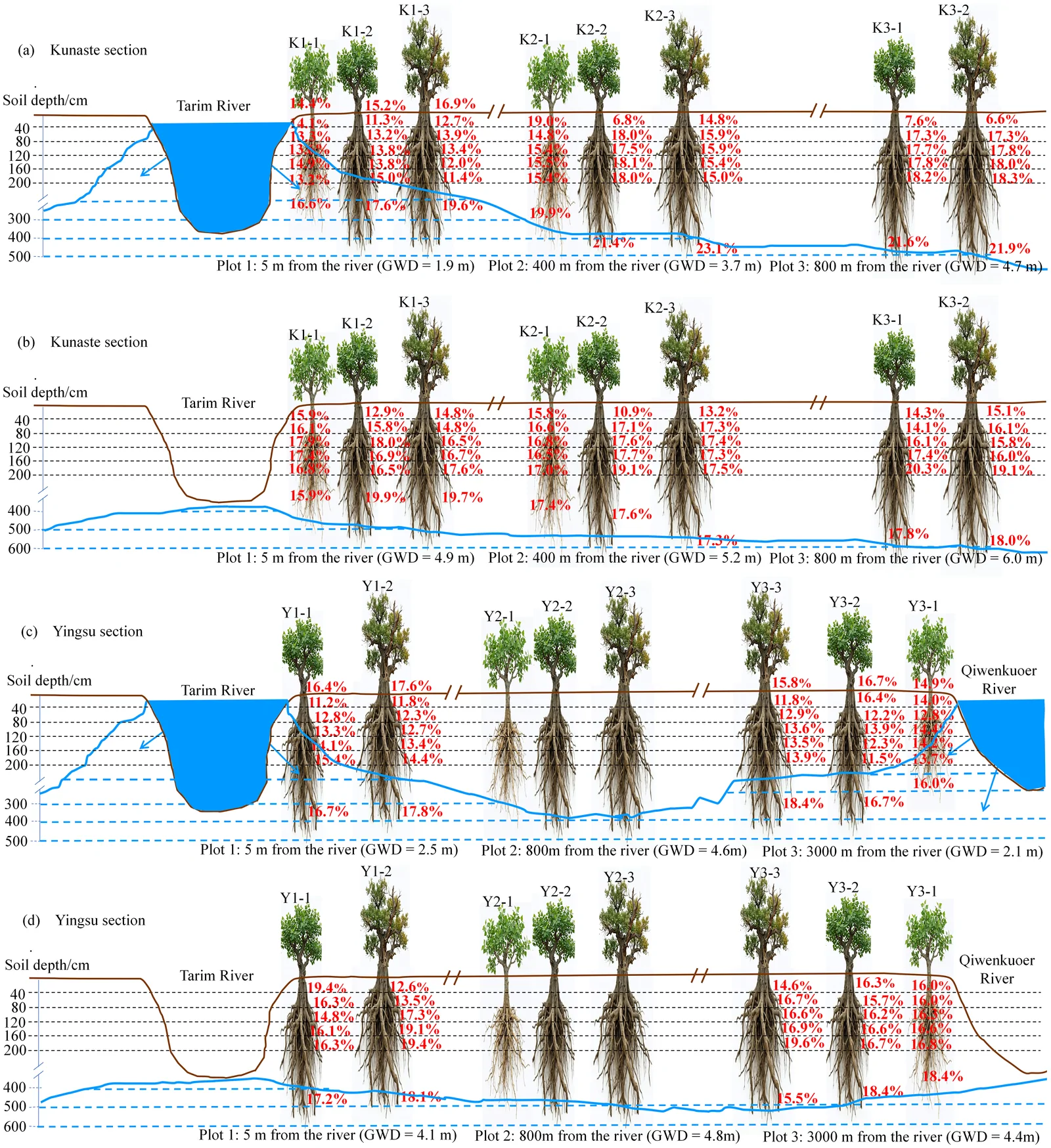
MixSIAR posterior mean estimates of water source contributions to *P. euphratica* xylem water at the DW and F sections during EWC and non-EWC periods. **(a, b)** show the DW section during EWC and non-EWC periods, respectively; **(c, d)** show the F section during the corresponding periods.

At DW, groundwater depth ranged from 1.9 to 4.7 m across the three plots during EWC (**Figure 3a**). Posterior mean contributions ranged from 16.6 to 23.1% for groundwater and from 6.6 to 19.0% for individual 40-cm soil layers. At Plot 1, where river water was included as a potential source, its contribution ranged from 14.4 to 16.9% among age classes. During the non-EWC period, groundwater depth ranged from 4.9 to 6.0 m (**Figure 3b**), with posterior mean contributions of 15.9–19.9% from groundwater and 10.9–20.3% from individual soil layers. The 160–200 cm soil layer contributed 16.5–20.3% during the non-EWC period and 12.7–18.3% during EWC.

At F Plot 2, the volume of extracted soil water was insufficient for δ^2^H and δ^18^O analysis; therefore, the isotopic composition of the soil water sources could not be characterized, and source contributions were not estimated for this plot. During EWC, groundwater depths at Plots 1 and 3 were 2.5 and 2.1 m, respectively (**Figure 3c**). Across these plots, posterior mean contributions were 16.0–18.4% for groundwater, 14.9–17.6% for river water, and 11.2–16.4% for individual 40-cm soil layers. During the non-EWC period, groundwater depths at Plots 1 and 3 were 4.1 and 4.4 m, respectively (**Figure 3d**), with posterior mean contributions of 15.5–18.4% from groundwater and 12.6–19.6% from individual soil layers. The 95% CrIs were generally broad and overlapped across sources and age classes (**Supplementary Table S7**). Overall, water source partitioning varied across hydrological periods and groundwater depth gradients, whereas age-related differences were less consistent and were accompanied by broad, overlapping posterior credible intervals.

### 3.3 Variation in iWUE in relation to groundwater depth

#### 3.3.1 Variation in iWUE along the groundwater depth gradient

Leaf δ^13^C-derived iWUE showed substantial variation across the groundwater depth gradient during both hydrological periods (**Figures 4a, b**). Groundwater depth ranged from 1.9 to 6.9 m during EWC and from 2.35 to 6.20 m during non-EWC periods, with most observations between 4.0 and 6.0 m. Within this range, iWUE spanned 28.80–88.29 μmol mol^−1^ during EWC and 27.94–90.43 μmol mol^−1^ during non-EWC periods, with considerable overlap among age classes. Quadratic fits explained little of the variation in iWUE, with *R*² values of 0.01–0.08 during EWC and 0.05–0.25 during non-EWC periods; none of the relationships was significant (*P* > 0.05). Substantial variation in iWUE therefore remained among trees at similar groundwater depths, indicating limited explanatory power of groundwater depth alone.

**Figure 4:**
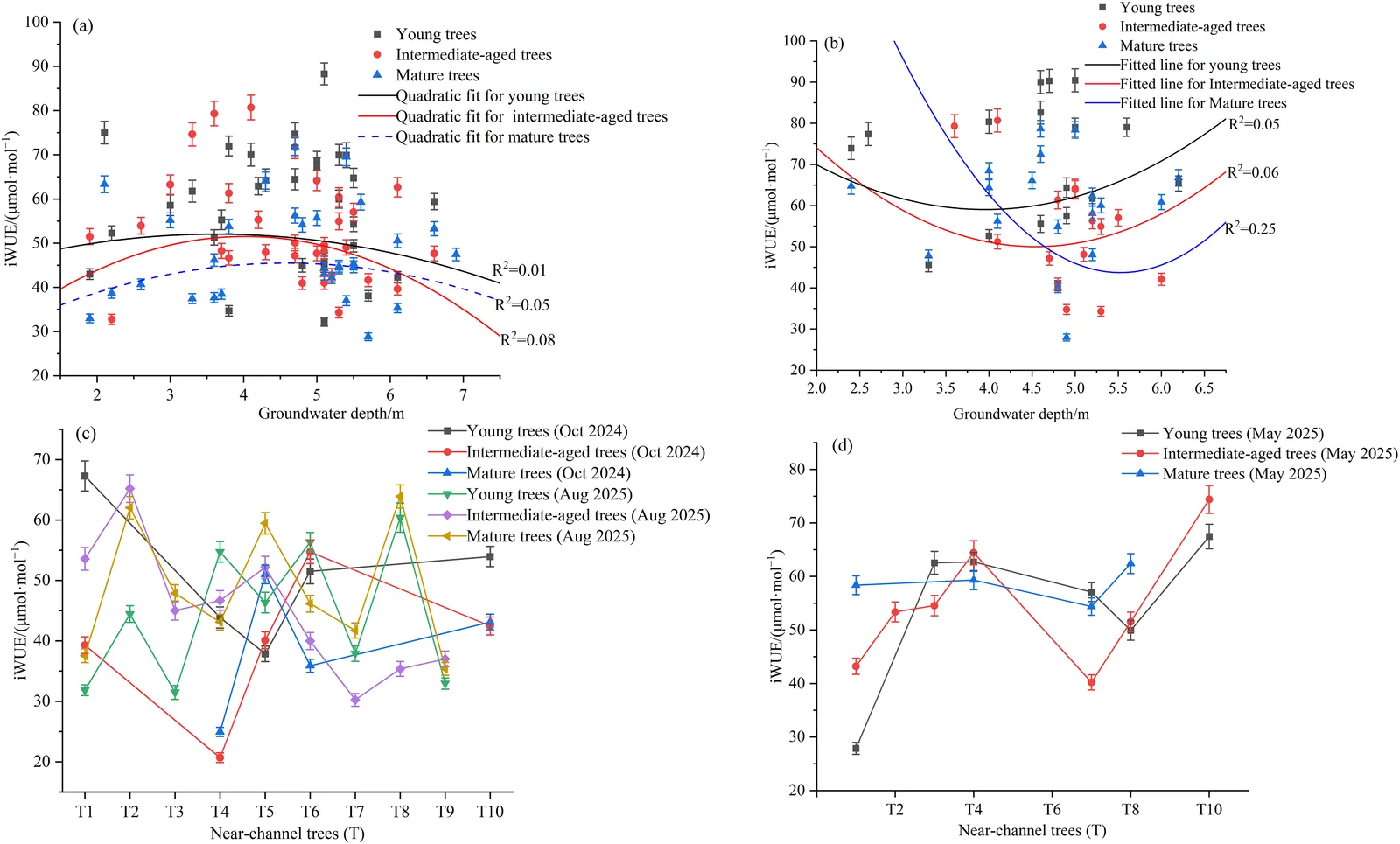
Changes in iWUE of *P. euphratica* trees of different age classes along the groundwater-depth gradient. **(a)** Groundwater-depth gradient during EWC; **(b)** groundwater-depth gradient during non-EWC; **(c)** near-channel trees during EWC; **(d)** near-channel trees during non-EWC.

#### 3.3.2 Temporal variation in iWUE across age classes

Across the five sampling campaigns, mean leaf δ¹³C-derived iWUE was 58.29 ± 1.91 μmol mol^−1^ in young trees (*n* = 67), 53.08 ± 1.51 μmol mol^−1^ in middle-aged trees (*n* = 71), and 51.37 ± 1.56 μmol mol^−1^ in mature trees (*n* = 65). Individual iWUE values ranged from 27.88 to 90.43 μmol mol^−1^ in young trees, from 20.67 to 80.69 μmol mol^−1^ in middle-aged trees, and from 24.93 to 78.68 μmol mol^−1^ in mature trees. Age class (*F*_2,196_ = 5.08, *P* = 0.007) and sampling period (*F*_4,196_ = 9.54, *P* < 0.001) were both significantly associated with iWUE, whereas the age class × sampling period interaction was not significant (*F*_8,188_ = 0.28, *P* = 0.973). Sampling period had a larger effect size than age class (partial η^2^ = 0.163 and 0.049, respectively).Model diagnostics are shown in **Supplementary Figures S4a, b**.

Across all tree-level samples, mean iWUE was 50.77, 54.46, 51.14, 63.16, and 50.13 μmol mol^−1^ in September 2023, July 2024, October 2024, May 2025, and August 2025, respectively. Mean iWUE was highest in young trees in each campaign, whereas the relative ranking of middle-aged and mature trees varied among campaigns. All three age classes had their highest mean iWUE in May 2025, reaching 66.52, 60.53, and 62.45 μmol mol^−1^ in young, middle-aged, and mature trees, respectively. The corresponding means in August 2025 were 52.96, 49.46, and 47.86 μmol mol^−1^. At near-channel sites, mean iWUE increased from 43.33 μmol mol^−1^ in October 2024 to 55.51 μmol mol^−1^ in May 2025 and then decreased to 45.88 μmol mol^−1^ in August 2025 (**Figures 4c, d**). These values were lower than the corresponding means across the five monitoring sections (55.35, 66.58, and 52.48 μmol mol^−1^, respectively).

For the 145 tree-level samples with corresponding GWD measurements, adding a common GWD term to the model containing age class and sampling period did not improve model fit (*R*^2^ = 0.268–0.269; *F*_1,137_ = 0.29, *P* = 0.589). Adding the GWD × sampling period interaction significantly improved model fit (*F*_4,133_ = 4.12, *P* = 0.004), increasing *R*^2^ to 0.350. By contrast, the GWD × age class interaction was not significant (*F*_2,131_ = 2.09, *P* = 0.127). Thus, the association between GWD and iWUE varied among sampling periods but showed no consistent age-dependent pattern. Model diagnostics are provided in **Supplementary Figure S3**.

#### 3.3.3 Associations of groundwater depth with water-source contributions and iWUE

When data from September 2023 and July 2024 were pooled, the contribution of shallow soil water showed a significant quadratic relationship with GWD (*R*^2^ = 0.433, *P* < 0.01), reaching a fitted maximum at 4.42 m before declining (**Figure 5c**). The contribution of deep soil water also showed a significant quadratic relationship with GWD (*R*^2^ = 0.766, *P* < 0.001), increasing over most of the observed range and reaching a fitted maximum at 5.96 m (**Figure 5b**). In contrast, groundwater contribution was not significantly related to GWD (*R*^2^ = 0.152, *P* = 0.151) (**Figure 5a**). The quadratic relationship between GWD and leaf δ^13^C-derived iWUE was also not significant (R^2^ = 0.100, *P* = 0.298), with the fitted curve reaching a maximum at approximately 6.0 m (**Figure 5d**).

**Figure 5:**
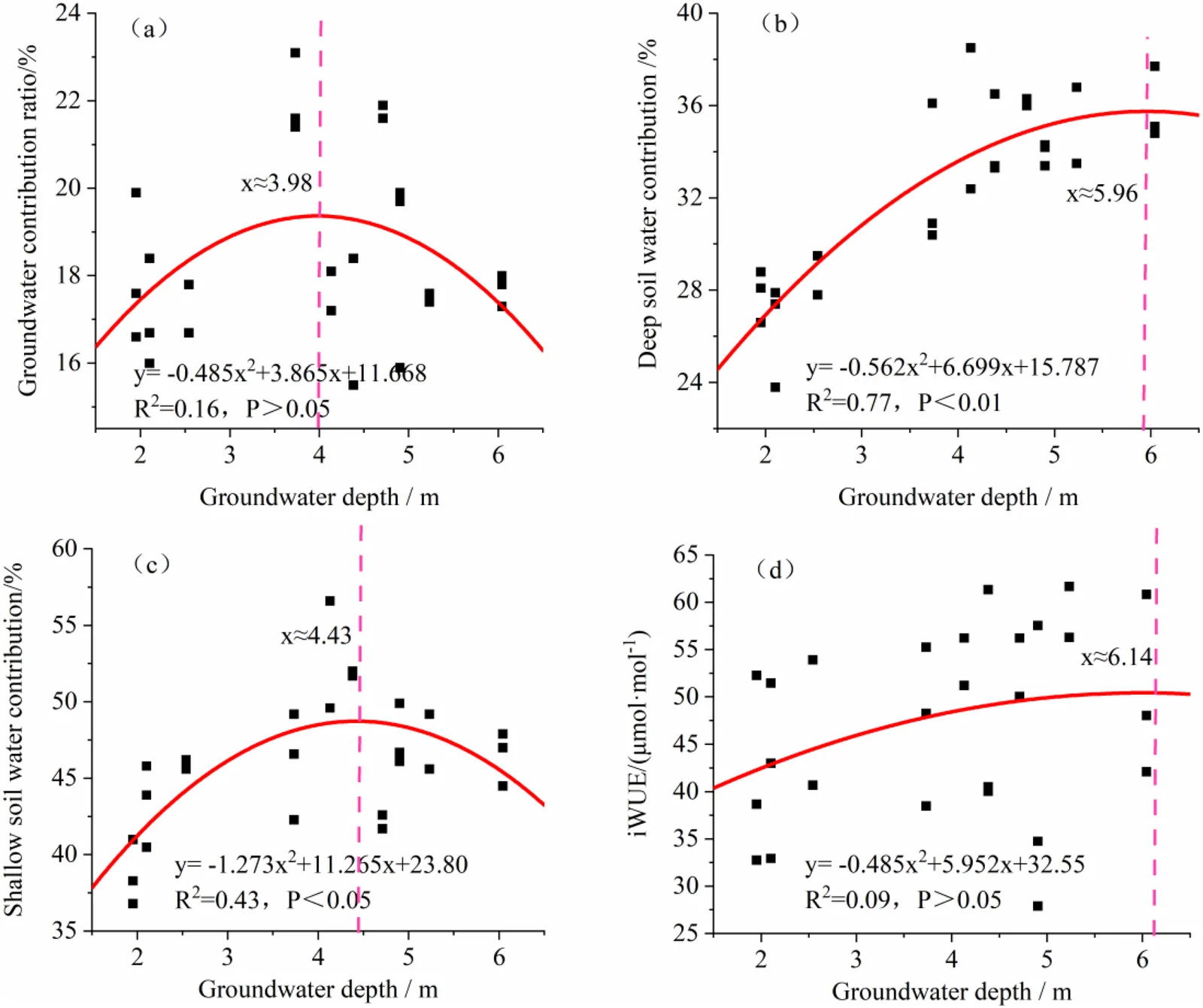
Relationships of groundwater depth with water-source contributions and iWUE in *P. euphratica*. **(a–d)** show the relationships of groundwater depth with the estimated proportional contributions of groundwater, deep soil water, and shallow soil water, and with iWUE, respectively.

### 3.4 Associations of iWUE with water-source use and ecophysiological variables

#### 3.4.1 Relationships between water source contributions and iWUE

For the correlation analysis, MixSIAR posterior mean contributions were grouped into relatively stable sources, comprising groundwater and soil water at 120–200 cm, and relatively dynamic sources, comprising river water and soil water at 0–120 cm during EWC and soil water at 0–120 cm during non-EWC. During the September 2023 EWC campaign, relationships between source contributions and leaf δ^13^C-derived iWUE varied among age classes (**Figures 6a–c**). In young trees, iWUE was positively correlated with the contribution of relatively stable sources (*r* = 0.75, *P* > 0.05) and negatively correlated with that of relatively dynamic sources (*r* = −0.75, *P* > 0.05). In middle-aged trees, iWUE was negatively correlated with relatively stable sources (*r* = −0.71, *P* < 0.05) and positively correlated with relatively dynamic sources (*r* = 0.73, *P* < 0.05). In mature trees, iWUE was positively correlated with relatively stable sources (*r* = 0.85, *P* < 0.05), while the correlation with relatively dynamic sources was negative (*r* = −0.80, *P* > 0.05).

**Figure 6:**
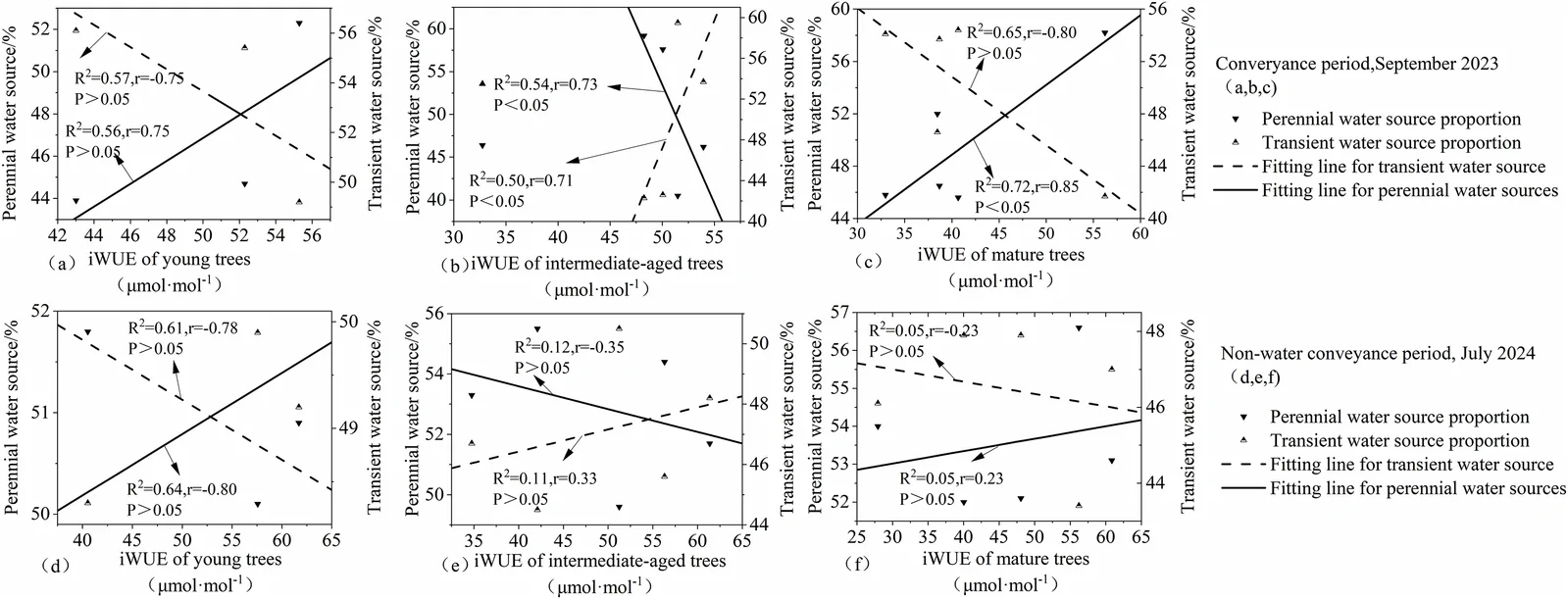
Relationships between MixSIAR posterior mean estimates of water-source contributions and leaf δ^13^C-derived iWUE in *P. euphratica* at the DW and F sections. **(a–c)** show young, middle-aged, and mature trees, respectively, during the September 2023 EWC campaign; **(d–f)** show the corresponding age classes during the July 2024 non-EWC campaign.

During the July 2024 non-EWC campaign, none of the correlations between posterior mean source contributions and iWUE was significant (**Figures 6d–f**; all *P* > 0.05). In young trees, iWUE was positively correlated with relatively stable sources (*r* = 0.80) and negatively correlated with relatively dynamic sources (*r* = −0.78). In middle-aged trees, the corresponding correlations were negative for relatively stable sources (*r* = −0.35) and positive for relatively dynamic sources (*r* = 0.33). In mature trees, the correlations were positive for relatively stable sources (*r* = 0.23) and negative for relatively dynamic sources (*r* = −0.23).

#### 3.4.2 Relationships between iWUE and environmental variables and leaf traits

The relationships between leaf δ^13^C-derived iWUE and the measured environmental and physiological variables varied among sampling periods (**Figure 7**). GWD was positively correlated with iWUE in September 2023 (*r* = 0.71, *P* < 0.05) but negatively correlated in October 2024 (*r* = −0.39, *P* < 0.05). No significant GWD–iWUE relationship was observed in July 2024 (*r* = 0.083, *P* > 0.05) or May 2025 (*r* = 0.043, *P* > 0.05).

**Figure 7:**
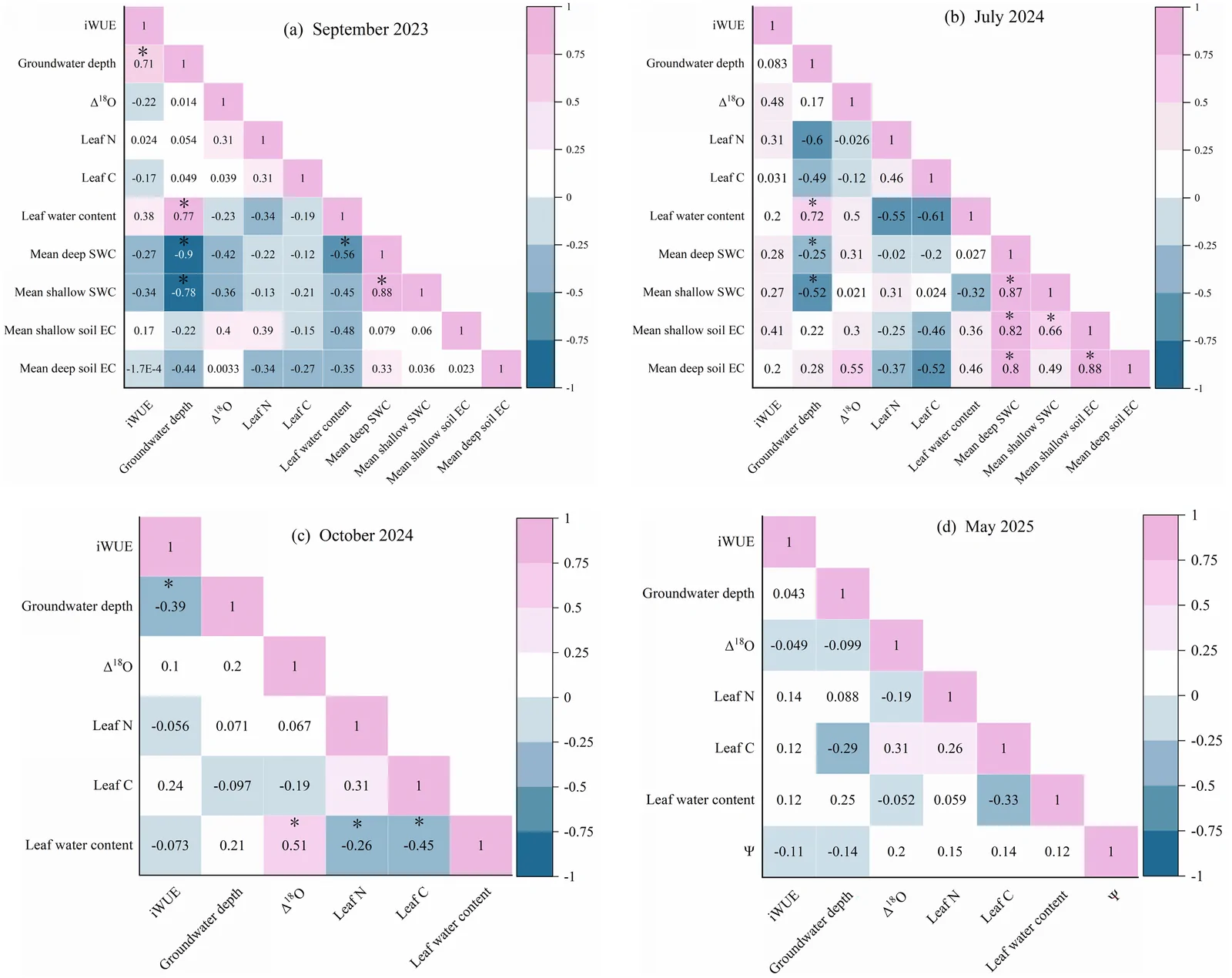
Pearson correlation matrices for leaf δ^13^C-derived iWUE and measured environmental and physiological variables in *P. euphratica*. **(a–d)** correspond to September 2023, July 2024, October 2024, and May 2025, respectively. Cell values indicate Pearson correlation coefficients (r), and asterisks denote P < 0.05.

No significant correlations were found between iWUE and Δ^18^O, leaf N content, leaf C content, or leaf water content in any of the four sampling periods (all *P* > 0.05). Similarly, shallow SWC, deep SWC, and soil EC were not significantly correlated with iWUE in September 2023 or July 2024, and leaf water potential (Ψ) was not significantly correlated with iWUE in May 2025. Thus, apart from the sampling-period-specific correlations with GWD, no measured variable showed a consistently significant relationship with iWUE across the sampling periods examined.

## 4 Discussion

### 4.1 Hydrological and age-related variation in water source use

Water source use differed between the EWC and non-EWC campaigns. During EWC, MixSIAR posterior mean estimates indicated contributions from groundwater, river water, and soil water to *P. euphratica* xylem water; during non-EWC, river water was unavailable and several deeper soil layers made relatively greater contributions. Wan et al. (2024b) documented shifts in water source use by *P. euphratica* across flood stages in the Taklimakan Desert as surface-water and groundwater conditions changed, supporting the hydrological sensitivity observed here. Across the pooled September 2023 and July 2024 observations, contributions from shallow and deep soil water showed significant quadratic relationships with GWD, whereas groundwater contribution did not. Li et al. (2022) also reported nonlinear changes in contributions from upper and deep-water sources along a gradient in water-table depth in riparian *Salix babylonica*. In the present dataset, variation along the GWD gradient was therefore expressed mainly through changes in the relative use of soil water at different depths rather than through a monotonic increase in groundwater contribution. The source-partitioning results did not indicate a strong separation between plant-used water and mobile water sources, because groundwater contributed to *P. euphratica* xylem water in both hydrological periods and river water also contributed during EWC. Although groundwater and river water were represented in the estimated source mixtures, the present sampling design did not distinguish mobile soil water from more tightly retained soil water. Therefore, our data do not allow a direct test of the “Two Water Worlds” hypothesis.

Age-related differences were most apparent during EWC. In the three EWC plots containing all age classes, posterior mean groundwater contribution increased from young to middle-aged and mature trees. Liu et al. (2015) found a comparable age-related pattern along the lower Heihe River, where young *P. euphratica* relied more on shallow soil water, whereas mature and over-mature trees used more water from deeper soil layers and groundwater. The same ordering was not maintained during non-EWC. Wan et al. (2024b) also reported relatively small differences in water source patterns among tree-size classes. Age-related differentiation in water source use was therefore evident during EWC but weakened during non-EWC. These findings supported H1: water source partitioning differed between EWC and non-EWC periods and varied along the groundwater depth gradient, with age-related differences being more evident during EWC than during non-EWC periods.

Soil water was sampled to a maximum depth of 2 m, whereas GWD exceeded 4 m at many sites. Water in the unsampled vadose zone below 2 m was therefore not represented as a separate source in MixSIAR; any uptake from this interval could not be resolved independently, increasing uncertainty in the posterior source estimates (Rothfuss and Javaux, 2017). Source partitioning was also restricted to six plots at DW and F. Because vegetation responses to water supply vary spatially along the lower Tarim River (Song et al., 2024), the source estimates primarily characterize these two sampled sections, and their applicability elsewhere along the lower river remains to be tested with comparable source water sampling.

### 4.2 Age-related and temporal variation in iWUE and environmental correlates

Leaf δ^13^C-derived iWUE differed significantly among age classes and sampling periods, with young trees showing the highest mean values and sampling period having a larger effect than age class (partial η^2^ = 0.163 and 0.049, respectively). Age-related differences in water acquisition may partly underlie this pattern because young *P. euphratica* tend to rely more on shallow soil water, whereas mature trees have greater access to deep soil water and groundwater (Liu et al., 2015). However, higher WUE in mature trees has also been reported under different groundwater and flooding conditions (Wan et al., 2024b), indicating that age-related variation in iWUE depends on the hydrological conditions experienced by different age classes rather than following a fixed ontogenetic pattern.

The stronger temporal variation was accompanied by a period-dependent GWD–iWUE relationship. After age class and sampling period were accounted for, GWD had little additional explanatory power (*P* = 0.589), whereas the GWD × sampling-period interaction was significant (*P* = 0.004). GWD was positively related to iWUE in September 2023, negatively related in October 2024, and unrelated in July 2024 and May 2025 (**Figures 7a–d**). Because iWUE reflects the balance between carbon assimilation and stomatal conductance, changes in water availability need not produce a uniform directional response when these two processes change to different degrees. Wan et al. (2024a) similarly reported seasonal variation in the influence of groundwater depth on *P. euphratica* sap flow, while VPD and solar radiation were also important environmental controls. In the present study, sap flow at DW was not significantly related to either GWD or VPD in simple linear analyses (**Supplementary Figure S4**), further indicating that temporal variation in plant water use cannot be attributed to groundwater depth alone.

SWC and soil EC were not significantly related to iWUE in September 2023 or July 2024 (**Figures 7a, b**), although these variables are closely associated with water acquisition. In the same DW and F sampling system, Jiang et al. (2025) showed that soil moisture and salinity affected the proportions of water taken up from different sources. The present results further showed that source contribution–iWUE relationships varied among age classes during EWC and were nonsignificant during non-EWC (**Figure 6**). Soil water and salinity may therefore modify water source use without producing a proportional change in leaf-level iWUE, because subsequent carbon assimilation and stomatal regulation need not respond in parallel. Leaf-water Δ^18^O was not significantly related to iWUE. This may partly reflect the fact that leaf-water ^18^O enrichment is influenced not only by stomatal regulation but also by evaporative conditions and within-leaf water transport (Scheidegger et al., 2000; Barbour et al., 2004). Leaf water content was also unrelated to iWUE across the four campaigns, and leaf water potential showed no significant relationship with iWUE in May 2025 (**Figure 7**). These results indicate that variation in leaf δ^13^C-derived iWUE was not consistently associated with the measured indicators of leaf water status.

Because iWUE was estimated from bulk leaf δ^13^C, its interpretation depends on the assumptions and atmospheric inputs used in the isotope model. The use of global monthly C_a_ in place of local atmospheric CO_2_ measurements and a fixed atmospheric δ^13^C_a_ value introduces additional uncertainty in the absolute iWUE estimates. Sensitivity analysis using the available monthly background δ^13^C_a_ values altered the absolute estimates but preserved the age-class ranking within the three sampling periods for which these data were available (**Supplementary Table S5**). Finite mesophyll conductance and post-photosynthetic fractionation can also affect δ^13^C-based iWUE estimates (Ma et al., 2021; Gimeno et al., 2021; Ma et al., 2023). Because these processes were not quantified here, we emphasize relative differences among age classes and sampling periods rather than the precise absolute values of the estimated iWUE.

### 4.3 Water source use and iWUE across age classes and hydrological periods

During EWC, iWUE was negatively related to the contribution of relatively stable water sources in middle-aged trees but positively related in mature trees, whereas no significant relationship was detected in young trees. During non-EWC, none of these relationships was significant (**Figure 6**). Greater use of relatively stable water sources was therefore not consistently associated with higher iWUE. Positive associations between deeper water use and WUE have been reported in subtropical shrubs and karst woody species (Jiang et al., 2020; Ding et al., 2021), while leaf δ^13^C increased with the contribution of deep water in riparian *Salix babylonica* (Li et al., 2022). By contrast, studies summarized by Bachofen et al. (2024) also include cases in which deeper water uptake was associated with lower WUE. Water uptake depth therefore does not show a consistent relationship with WUE across woody plants.

The absence of a consistent relationship can be understood from the physiological basis of iWUE. Stable-isotope source partitioning characterizes the origin of plant water uptake, whereas iWUE reflects carbon assimilation relative to stomatal conductance (Rothfuss and Javaux, 2017; Farquhar et al., 1989). Photosynthetic capacity and stomatal conductance can vary independently and thereby alter iWUE (Gilbert et al., 2011). Changes in water-source use therefore need not be accompanied by a similar change in iWUE. From the dry year to the wet year, mean water-table depth in riparian *S. babylonica* habitat decreased from 2.7 to 1.7 m, while the mean contribution of river water to transpiration increased from 16.8% to 23.8% and leaf δ^13^C decreased from −27.7‰ to −29.7‰. Li et al. (2023) suggested that greater water availability in the wet year probably promoted stomatal opening and transpiration, contributing to the lower leaf δ^13^C. In *Salix psammophila*, the main water source shifted from shallow to middle soil layers during a dry year, accompanied by greater use of deep soil water and an approximately 10% decrease in iWUE (Wang et al., 2025).

Age-related differences in water-source use have also been reported for *P. euphratica*. Young trees along the lower Heihe River relied mainly on shallow soil water, whereas mature and over-mature trees used more water from below 100 cm, derived largely from groundwater (Liu et al., 2015). Water-source use also changed across flood stages in the Taklimakan Desert: *P. euphratica* used flood water during flooding and subsequently returned to greater use of deep soil water and water from layers close to the groundwater table, while WUE varied with growth stage and water conditions (Wan et al., 2024b). In the present study, river water contributed during EWC, whereas deeper soil layers made relatively greater contributions during non-EWC. Nevertheless, significant relationships between relatively stable water sources and iWUE occurred only in middle-aged and mature trees during EWC. Because photosynthetic carbon assimilation and stomatal conductance were not measured concurrently, the physiological mechanisms underlying the age-specific source–iWUE relationships cannot be resolved from the present data. Nevertheless, the observed source–iWUE patterns were consistent with H2: the relationships differed among age classes during EWC, whereas no significant relationships were detected during non-EWC periods. These results also indicate that greater reliance on relatively stable water sources was not consistently associated with higher iWUE.

### 4.4 Potential groundwater depth range for *P. euphratica* and study limitations

Shallow soil-water contribution increased with groundwater depth to a fitted maximum at approximately 4.42 m and declined thereafter (P < 0.01), whereas deep soil-water contribution continued to increase to a fitted maximum at approximately 5.96 m (P < 0.001; **Figure 5**). The fitted iWUE curve peaked at approximately 6.0 m, but its relationship with groundwater depth was not significant and was therefore considered only as supporting evidence. Based on these changes in water-source contributions, we identify 4.5–6.0 m as a preliminary groundwater depth range associated with shifts in water uptake by *P. euphratica*.

The 4.5–6.0 m range overlaps with several previously reported groundwater depth thresholds for *P. euphratica*, although these thresholds represent different biological responses. At the leaf scale, net photosynthetic rate remained relatively stable between 4.2 and 6.8 m, whereas stomatal conductance and transpiration declined at 6.8 m while iWUE increased, suggesting that higher iWUE under deeper groundwater conditions may reflect stronger stomatal regulation rather than improved water availability (Chen et al., 2012). At longer timescales, tree-ring growth showed clear age dependence, with critical groundwater depths increasing from approximately 4.0 m in young trees and 5.0–5.4 m in near-mature trees to 6.9 m in mature trees and 7.8 m in over-mature trees (Ling et al., 2015). At the vegetation scale, Chen et al. (2015) identified 2–4 m as an appropriate ecological groundwater depth and approximately 6 m as a threshold for local vegetation in the lower Tarim River. Li et al. (2023) further showed that threshold estimates varied with the response metric: tree-ring N and P responses indicated a critical depth of 5.20–5.31 m, whereas EVI loss exhibited critical changes at 3.85, 6.18–6.75, and 7.89 m. These findings indicate that groundwater depth thresholds depend on the response variable, timescale, and level of biological organization. The 4.5–6.0 m range identified here specifically reflects a shift in water uptake from shallow to deeper soil water and therefore differs from thresholds derived from gas exchange, radial growth, species diversity, or canopy responses.

Interpretation of this range is constrained by the spatial and vertical resolution of water-source sampling. Water source partitioning was conducted only at the DW and F sections during two sampling campaigns, limiting representation of spatial heterogeneity along the lower Tarim River. Soil water was sampled only to 2 m; where groundwater occurred below this depth, vadose-zone water between 2 m and the water table was not included as a separate source in MixSIAR and therefore could not be resolved independently. Broad posterior credible intervals for some source estimates further increase uncertainty in the fitted transition points. In addition, the relationship between groundwater depth and iWUE was not significant, and continuous meteorological observations were available only at DW. The 4.5–6.0 m interval should therefore be regarded as a preliminary reference range for shifts in water uptake under the conditions examined here. Its robustness and spatial applicability require further evaluation using deeper vadose-zone sampling, broader spatial replication, and longer-term observations.

## 5 Conclusions

Groundwater depth differed significantly between EWC and non-EWC periods in the lower Tarim River. Mean groundwater depth was 4.65 ± 0.96 m during EWC and 4.92 ± 0.76 m during non-EWC periods (*P* = 0.001), with the magnitude of the difference varying among sections. At the DW and F sections, MixSIAR posterior estimates showed shifts in water source use between the two hydrological periods. During EWC, posterior mean contributions ranged from 16.0 to 23.1% for groundwater, from 14.4 to 17.6% for river water, and from 6.6 to 19.0% for individual soil layers. During non-EWC periods, contributions from individual soil layers ranged from 10.9 to 20.3%, with relatively greater contributions from deeper soil layers.

Leaf δ^13^C-derived iWUE differed significantly among age classes and sampling periods, averaging 58.29 ± 1.91 μmol mol^−1^ in young trees, 53.08 ± 1.51 μmol mol^−1^ in middle-aged trees, and 51.37 ± 1.56 μmol mol^−1^ in mature trees. Sampling period explained more variation in iWUE than age class, and the relationship between GWD and iWUE varied among sampling periods. Relationships between water source contributions and iWUE differed among age classes during EWC, whereas no significant relationships were detected during non-EWC periods.

Based primarily on changes in shallow and deep soil water contributions along the groundwater depth gradient, 4.5–6.0 m was identified as a preliminary groundwater depth range associated with shifts in water source use by *P. euphratica*. This estimate is constrained by source partitioning at only the DW and F sections and soil water sampling limited to 2 m. Broader spatial replication, deeper vadose-zone sampling, and longer-term observations are needed to assess its wider applicability.

## Data Availability

The raw data supporting the conclusions of this article will be made available by the authors, without undue reservation.
