# Supplementary material for: Water use strategies of Populus euphratica across age classes along groundwater depth gradients in the lower Tarim River
Source: Front Plant Sci. 2026 Oct 5;17:1964884. doi: 10.3389/fpls.2026.1964884 (PMC13677497; doi:10.3389/fpls.2026.1964884)
Supplement: Supplementary file 1 [file DataSheet1.docx]

**Fig. S1. Representative field conditions across EWC and non-EWC stages in the lower Tarim River.** (a) EWC period; (b) early non-EWC period; (c) middle non-EWC period; (d) late non-EWC period.

**Fig. S2. Annual variations in meteorological conditions at the DW section.** (a) Relative humidity and air temperature; (b) wind speed; (c) total solar radiation.

**Fig. S3. Diagnostic plots for the general linear models of leaf δ^13^C-derived iWUE.** Panels (a) and (b) show internally studentized residuals plotted against fitted values and the normal Q–Q plot of the same residuals, respectively, for the model including age class and sampling period (n = 203). Panels (c) and (d) show the corresponding diagnostics for the final model including age class, sampling period, mean-centered groundwater depth (GWD), and the GWD × sampling-period interaction (n = 145).

**Fig. S4. Correlations of sap flow monitored at 1-h intervals with groundwater depth and VPD.**Panels (a), (b), and (c) represent young, middle-aged, and mature *P. euphratica*, respectively.

**Table S1. Number of *P. euphratica* samples by sampling period, sampling section/site, and age class.** Young: DBH ≤ 10 cm; middle-aged: 10 < DBH ≤ 20 cm; mature: DBH > 20 cm. T-coded samples denote trees collected at near-channel sites along the Tarim River and are listed separately from the five fixed monitoring sections.

| **Sampling period** | **Hydrological period** | **Sampling section/site** | **Young** | **Middle-aged** | **Mature** | **Total** |
| --- | --- | --- | --- | --- | --- | --- |
| Sep 2023 | EWC | Kunasite (DW) | 2 | 3 | 3 | 8 |
|  |  | Yingsu (F) | 2 | 3 | 3 | 8 |
| Jul 2024 | non-EWC | Kunasite (DW) | 2 | 3 | 3 | 8 |
|  |  | Yingsu (F) | 2 | 3 | 3 | 8 |
| Oct 2024 | EWC | Kunasite (DW) | 1 | 1 | 1 | 3 |
|  |  | Yingsu (F) | 4 | 4 | 1 | 9 |
|  |  | Kardeyi (G) | 1 | 1 | 0 | 2 |
|  |  | Alagan (LH) | 2 | 3 | 2 | 7 |
|  |  | Yiganbujima (I) | 1 | 0 | 4 | 5 |
|  |  | Tarim River near-channel sites (T) | 5 | 5 | 4 | 14 |
| May 2025 | non-EWC | Kunasite (DW) | 2 | 3 | 2 | 7 |
|  |  | Yingsu (F) | 3 | 2 | 3 | 8 |
|  |  | Kardeyi (G) | 3 | 3 | 0 | 6 |
|  |  | Alagan (LH) | 3 | 5 | 4 | 12 |
|  |  | Yiganbujima (I) | 2 | 0 | 3 | 5 |
|  |  | Tarim River near-channel sites (T) | 6 | 7 | 4 | 17 |
| Aug 2025 | EWC | Kunasite (DW) | 2 | 2 | 2 | 6 |
|  |  | Yingsu (F) | 4 | 4 | 3 | 11 |
|  |  | Kardeyi (G) | 2 | 3 | 3 | 8 |
|  |  | Alagan (LH) | 6 | 5 | 5 | 16 |
|  |  | Yiganbujima (I) | 3 | 2 | 3 | 8 |
|  |  | Tarim River near-channel sites (T) | 9 | 9 | 9 | 27 |

**Table S2. Basic information of the sample plot**. “±” in the table means “mean±standard deviation”. The letters in the sample number represent the section number of the sampling point, the first digit represents the sample plot number, and the second digit represents the sample trees of different ages in the sample plot.

| **Sections**  **name** | **Sample plot number** | **Sample tree number** | **Distance from shore/m** | **Groundwater depth/m** | | **Tree age** | **Tree height/m** | **Crown width/m**  **(North-South × East-West)** | **DBH**  **(diameterat breastheight)/cm** |
| --- | --- | --- | --- | --- | --- | --- | --- | --- | --- |
|  |  |  |  | Conveyance  period | Non-conveyance  period |  |  |  |  |
| Kunaste section | Plot 1 | K1-1 | 0~5 | 1.9 | 4.9 | Young | 1.0±0.4 | (1.4±0.4)×(1.5±0.3) | 5.6±0.7 |
|  |  | K1-2 |  |  |  | Middle-aged | 7.2±1.0 | (3.3±0.5)×(2.9±0.4) | 15.6±1.3 |
|  |  | K1-3 |  |  |  | Mature | 9.7±0.9 | (4.6±0.5)×(4.3±0.6) | 23.6±1.1 |
|  | Plot 2 | K2-1 | 200~205 | 3.7 | 5.2 | Young | 1.8±1.0 | (2.0±0.4)×(1.8±0.2) | 4.9±0.7 |
|  |  | K2-2 |  |  |  | Middle-aged | 5.3±1.2 | (3.2±0.7)×(3.4±0.6) | 16.2±1.0 |
|  |  | K2-3 |  |  |  | Mature | 8.8±0.7 | (8.0±0.8)×(7.8±0.7) | 54.0±2.1 |
|  | Plot 3 | K3-1 | 400~405 | 4.7 | 6.0 | Middle-aged | 7.8±1.5 | (2.5±0.4)×(2.3±0.4) | 14.5±1.0 |
|  |  | K3-2 |  |  |  | Mature | 10.3±0.8 | (3.6±0.4)×(3.7±0.4) | 51.0±1.7 |
| Yingsu section | Plot 1 | Y1-1 | 0~5 | 2.5 | 4.1 | Middle-aged | 5.7±1.3 | (4.2±0.4)×(4.1±0.6) | 10.1±0.2 |
|  |  | Y1-2 |  |  |  | Mature | 10.6±0.7 | (6.7±0.2)×(6.7±0.3) | 24.5±1.8 |
|  | Plot 2 | Y2-1 | 800~805 | 4.6 | 4.8 | Young | 4.1±1.0 | (2.3±0.5)×(2.2±0.6) | 7.4±0.6 |
|  |  | Y2-2 |  |  |  | Middle-aged | 5.8±0.7 | (3.1±0.3)×(3.4±0.3) | 15.1±1.7 |
|  |  | Y2-3 |  |  |  | Mature | 7.9±0.3 | (3.6±0.5)×(3.7±0.5) | 25.5±0.8 |
|  | Plot 3 | Y3-1 | 2995~3000 | 2.1 | 4.4 | Young | 1.3±0.4 | (1.8±0.3)×(1.9±0.4) | 3.4±1.2 |
|  |  | Y3-2 |  |  |  | Middle-aged | 6.6±0.5 | (3.5±0.3)×(3.6±0.3) | 17.5±0.9 |
|  |  | Y3-3 |  |  |  | Mature | 8.4±0.6 | (4.2±0.3)×(4.2±0.1) | 24.4±1.0 |

**Table S3. Groundwater monitoring well information and corresponding changes in groundwater depth.** Blank values in the table indicate groundwater depth data that were not measured during the sampling period.

| **Section Name** | **Sample Point ID** | **Longitude** | **Latitude** | **Elevation/m** | **Groundwater Depth in Sep 2023/m** | **Groundwater Depth in Jul 2024/m** | **Groundwater Depth in Oct 2024/m** | **Groundwater Depth in May 2025/m** | **Groundwater Depth in Aug 2025/m** | **Distance from River/m** |
| --- | --- | --- | --- | --- | --- | --- | --- | --- | --- | --- |
| Kunaste Section | DW1 | 87°51'48.09" | 40°26'46.33" | 776.2 | 2.2 | 4.9 | 3.6 | 4.0 | 3.9 | 50 |
|  | DW2 | 87°51'51.99" | 40°26'46.15" | 776.9 | 3.7 | 5.2 | 3.7 | 4.0 | 4.0 | 100 |
|  | DW3 | 87°51'58.04" | 40°26'45.22" | 770.0 | 4.7 | 6.0 | 5.5 | 5.2 | 5.3 | 200 |
|  | DW4 | 87°52'6.65" | 40°26'45.35" | 773.5 |  | 3.9 | 3.2 | 2.6 | 3.0 | 300 |
| Yingsu Section | F1 | 87°56'27.24" | 40°25'55.10" | 793.4 |  |  |  | 4.8 | 3.3 | 50 |
|  | F2 | 87°56'27.41" | 40°25'50.35" | 769.3 | 2.0 | 4.4 | 2.7 | 3.6 | 3.8 | 150 |
|  | F3 | 87°56'27.38" | 40°25'43.92" | 774.23 | 3.5 | 4.6 | 3.1 | 3.8 | 4.5 | 300 |
|  | F4 | 87°56'27.56" | 40°25'35.84" | 769.3 | 4.3 | 5.0 | 3.9 | 4.2 | 4.9 | 500 |
|  | F5 | 87°56'27.59" | 40°25'26.14" | 783.6 | 3.7 |  |  | 4.7 | 5.0 | 600 |
|  | F6 | 87°56'27.57" | 40°24'33.56" | 771.3 | 4.7 | 5.7 | 3.9 | 4.4 | 4.9 | 1650 |
|  | F7 | 87°56'27.72" | 40°24'23.82" | 767.7 | 4.8 | 4.8 | 5.2 | 4.4 | 5.1 | 1950 |
|  | F8 | 87°56'27.37" | 40°24'15.52" | 773.9 | 4.7 | 5.0 | 5.1 | 4.5 | 5.2 | 2250 |
|  | F10 | 87°56'27.49" | 40°24'4.06" | 767.2 | 5.2 |  |  | 6.2 | 6.6 | 2500 |
|  | F11 | 87°56'27.55" | 40°24'0.68" | 770.1 | 2.6 | 4.1 | 3.6 | 3.3 | 4.3 | 2600 |
|  | F12 | 87°56'28.35" | 40°25'13.03" | 772.1 |  | 5.1 | 4.7 | 4.7 | 5.0 | 750 |
|  | F13 | 87°56'28.38" | 40°25'0.01" | 773.2 |  | 5.0 | 4.1 | 4.7 | 5.1 | 1050 |
|  | F14 | 87°56'28.80" | 40°24'44.27" | 772.8 |  | 5.4 | 5.0 | 4.8 | 4.7 | 1350 |
| Kardayi Section | G1 | 88°10'27.60" | 40°22'18.99" | 767.7 |  | 5.4 | 4.5 | 5.0 | 3.8 | 50 |
|  | G2 | 88°10'24.04" | 40°22'17.18" | 766.9 |  | 5.2 | 4.5 | 4.9 | 3.9 | 150 |
|  | G3 | 88°10'18.76" | 40°22'14.51" | 766.5 |  | 5.5 | 5.0 | 5.1 | 4.4 | 300 |
|  | G4 | 88°10'11.67" | 40°22'10.77" | 768.1 |  | 5.5 | 5.3 | 5.4 | 5.3 | 500 |
|  | G5 | 88°10'2.58" | 40°22'6.16" | 764.9 |  | 5.3 | 5.3 | 4.9 | 5.4 | 750 |
|  | G6 | 88°9'52.08" | 40°22'0.71" | 763.1 |  | 5.8 | 5.0 | 5.6 | 6.1 | 1050 |
| Alagan Section | LH1 | 88°21'34.31" | 40°8'42.82" | 758.6 |  | 6.2 | 4.9 | 5.2 | 5.3 | 50 |
|  | LH2 | 88°21'28.25" | 40°8'41.36" | 761.1 |  | 5.2 | 4.6 | 4.6 | 5.0 | 150 |
|  | LH3 | 88°21'24.46" | 40°8'40.06" | 759.2 |  | 5.6 | 4.7 | 4.6 | 5.1 | 300 |
|  | LH4 | 88°21'16.79" | 40°8'37.54" | 786.1 |  | 5.8 | 6.6 | 5.2 | 5.7 | 500 |
|  | LH5 | 88°21'6.49" | 40°8'33.48" | 759.9 |  | 5.1 | 5.0 | 4.6 | 5.1 | 750 |
|  | LH6 | 88°20'54.51" | 40°8'29.52" | 756.4 |  | 5.0 | 4.8 | 4.6 | 5.1 | 1050 |
|  | H1 | 88°22'28.21" | 40°7'38.96" | 759.9 |  | 5.8 | 5.4 | 5.3 | 5.5 | 50 |
|  | H2 | 88°22'24.16" | 40°7'39.07" | 772.7 |  | 6.0 | 5.3 | 5.2 | 6.0 | 150 |
|  | H3 | 88°22'17.81" | 40°7'38.76" | 758.4 |  | 5.5 | 5.6 | 5.3 | 6.1 | 300 |
| Yiganbujima Section | I1 | 88°22'47.69" | 39°47'14.05" | 746.9 |  | 3.0 | 2.1 | 2.4 | 3.3 | 50 |
|  | I2 | 88°22'42.80" | 39°47'12.92" | 745.9 |  | 4.6 | 3.3 | 4.0 | 4.2 | 150 |
|  | I3 | 88°22'37.59" | 39°47'12.05" | 747.2 |  | 4.3 | 3.2 | 3.4 | 4.2 | 300 |
|  | I4 | 88°22'29.34" | 39°47'10.67" | 760.1 |  | 5.5 | 3.6 | 4.8 | 5.4 | 500 |
|  | I5 | 88°22'18.99" | 39°47'8.71" | 742.4 |  | 5.5 | 4.8 | 5.0 | 5.5 | 750 |
|  | I6 | 88°22'6.83" | 39°47'6.39" | 746.1 |  | 5.4 | 5.6 | 5.3 | 5.5 | 1050 |
|  | I7 | 88°21'54.51" | 39°47'4.35" | 747.5 |  | 6.8 | 6.9 | 6.8 | 7.0 | 1350 |

**Table S4. Monthly atmospheric CO_2_ concentrations (*C_a_*) used for iWUE calculation**

| **Sampling period** | **Globally averaged monthly atmospheric CO_2_ concentration, *C_a_* (ppm)** |
| --- | --- |
| September 2023 | 417.18 |
| July 2024 | 421.60 |
| October 2024 | 422.34 |
| May 2025 | 426.90 |
| August 2025 | 423.04 |

**Table S5. Sensitivity of leaf δ^13^C-derived iWUE estimates to atmospheric δ^13^C values across *P. euphratica* age classes**. iWUE values are presented as mean ± SE. Monthly atmospheric δ^13^C_a_ values were obtained from the Scripps CO_2_ Program at Mauna Loa Observatory. Relative difference was calculated as ( [(iWUE_monthly_ –iWUE_-8‰_) / iWUE_-8‰_] × 100% ). SE, standard error.

| **Sampling period** | **Age class** | **Monthly δ^13^C_a_（‰）** | **iWUE with fixed −8‰**  **（μmol mol^−1^）** | **iWUE with monthly δ^13^C_a_**  **（μmol mol^−1^）** | **Relative difference (%)** |
| --- | --- | --- | --- | --- | --- |
| September 2023 | Young | −8.51 | 56.33±6.66 | 62.43±6.66 | 10.83 |
|  | Middle-aged | −8.51 | 51.36±5.08 | 57.46±5.08 | 11.88 |
|  | Mature | −8.51 | 46.48±6.00 | 52.59±6.00 | 13.15 |
| July 2024 | Young | −8.72 | 62.56±10.36 | 71.25±10.35 | 13.89 |
|  | Middle-aged | −8.72 | 51.60±4.63 | 60.31±4.62 | 16.88 |
|  | Mature | −8.72 | 51.92±7.14 | 60.62±7.14 | 16.76 |
| October 2024 | Young | −8.60 | 56.36±3.04 | 63.61±3.04 | 12.86 |
|  | Middle-aged | −8.60 | 50.28±4.43 | 57.54±4.42 | 14.44 |
|  | Mature | −8.60 | 46.26±3.01 | 53.32±3.01 | 15.26 |

**Table S6. δ^2^H and δ^18^O composition of potential water-source end-members used in the MixSIAR analyses.** Values are presented as mean ± SD. Soil water was grouped into five depth intervals (0–40, 40–80, 80–120, 120–160, and 160–200 cm) according to the source categories used in the MixSIAR analyses. For soil water, n denotes the number of source-isotope values included in the MixSIAR input datasets and used to calculate the reported mean; repeated river-water or groundwater signatures shared among age classes within the same plot were counted once. River water was included only when present as a potential source during the corresponding sampling period. EWC, ecological water conveyance; non-EWC, non-water-conveyance period; SD, standard deviation.

| **Sampling period** | **Sectiont** | **Water source** | **n** | **δ^2^H (‰)** | **δ^18^O (‰)** |
| --- | --- | --- | --- | --- | --- |
| September 2023  (EWC) | DW | 0–40 cm soil water | 8 | -42.95 ± 30.52 | -3.32 ± 4.26 |
|  |  | 40–80 cm soil water | 8 | -58.63 ± 4.39 | -4.19 ± 3.74 |
|  |  | 80–120 cm soil water | 8 | -60.62 ± 4.49 | -5.25 ± 3.70 |
|  |  | 120–160 cm soil water | 8 | -59.07 ± 9.15 | -6.28 ± 2.90 |
|  |  | 160–200 cm soil water | 8 | -62.26 ± 6.61 | -6.96 ± 2.80 |
|  |  | River water | 1 | -44.10 | -9.39 |
|  |  | Groundwater | 3 | -29.58 ± 2.70 | -9.53 ± 0.29 |
|  | F | 0–40 cm soil water | 5 | -47.35 ± 13.76 | -3.51 ± 6.42 |
|  |  | 40–80 cm soil water | 5 | -61.93 ± 7.84 | -8.13 ± 3.52 |
|  |  | 80–120 cm soil water | 5 | -51.47 ± 8.41 | -8.45 ± 2.16 |
|  |  | 120–160 cm soil water | 5 | -56.94 ± 6.84 | -10.39 ± 1.90 |
|  |  | 160–200 cm soil water | 5 | -62.38 ± 2.89 | -12.04 ± 1.79 |
|  |  | River water | 2 | -29.41 ± 1.87 | -9.49 ± 0.03 |
|  |  | Groundwater | 2 | -28.17 ± 0.86 | -9.68 ± 0.17 |
| July 2024  (non-EWC) | DW | 0–40 cm soil water | 8 | -44.35 ± 11.62 | -2.59 ± 5.33 |
|  |  | 40–80 cm soil water | 8 | -53.25 ± 9.71 | -4.45 ± 5.90 |
|  |  | 80–120 cm soil water | 8 | -53.87 ± 8.16 | -5.56 ± 5.98 |
|  |  | 120–160 cm soil water | 8 | -56.84 ± 5.17 | -6.90 ± 5.54 |
|  |  | 160–200 cm soil water | 8 | -56.29 ± 4.99 | -7.74 ± 5.41 |
|  |  | Groundwater | 3 | -59.38 ± 1.75 | -9.09 ± 0.77 |
|  | F | 0–40 cm soil water | 5 | -50.63 ± 6.26 | -1.99 ± 2.92 |
|  |  | 40–80 cm soil water | 5 | -60.78 ± 7.16 | -6.05 ± 1.72 |
|  |  | 80–120 cm soil water | 5 | -68.45 ± 5.54 | -9.16 ± 1.37 |
|  |  | 120–160 cm soil water | 5 | -66.89 ± 4.85 | -9.16 ± 1.57 |
|  |  | 160–200 cm soil water | 5 | -67.42 ± 4.98 | -9.36 ± 1.60 |
|  |  | Groundwater | 3 | -61.27 ± 1.01 | -10.36 ± 0.83 |

**Table S7. Posterior estimates and 95% credible intervals of water-source contributions to *P. euphratica* xylem water estimated using MixSIAR.** Values are posterior estimates from the MixSIAR analyses. The 95% Bayesian credible interval (CrI) is defined by the 2.5th and 97.5th percentiles of the posterior distribution. EWC, ecological water conveyance; non-EWC, non-water-conveyance period; CrI, credible interval.

| **Sampling period** | **Section** | **Plot** | **Age class** | **Water source** | **Posterior mean (%)** | **95% CrI (%)** |
| --- | --- | --- | --- | --- | --- | --- |
| September 2023  (EWC) | DW | Plot 1 | Young | 0–40 cm soil water | 14.1 | 0.4–44.4 |
|  |  |  |  | 40–80 cm soil water | 13.3 | 0.4–41.6 |
|  |  |  |  | 80–120 cm soil water | 13.6 | 0.5–42.6 |
|  |  |  |  | 120–160 cm soil water | 14.9 | 0.5–46.0 |
|  |  |  |  | 160–200 cm soil water | 13.2 | 0.4–42.3 |
|  |  |  |  | Groundwater | 16.6 | 0.6–51.0 |
|  |  |  |  | River water | 14.4 | 0.4–44.7 |
|  |  |  | Middle-aged | 0–40 cm soil water | 11.3 | 0.3–39.8 |
|  |  |  |  | 40–80 cm soil water | 13.2 | 0.4–43.6 |
|  |  |  |  | 80–120 cm soil water | 13.8 | 0.4–45.5 |
|  |  |  |  | 120–160 cm soil water | 13.8 | 0.4–45.2 |
|  |  |  |  | 160–200 cm soil water | 15.0 | 0.5–48.8 |
|  |  |  |  | Groundwater | 17.6 | 0.5–55.9 |
|  |  |  |  | River water | 15.2 | 0.5–49.9 |
|  |  |  | Mature | 0–40 cm soil water | 11.4 | 0.3–38.5 |
|  |  |  |  | 40–80 cm soil water | 12.0 | 0.3–40.3 |
|  |  |  |  | 80–120 cm soil water | 13.4 | 0.4–45.3 |
|  |  |  |  | 120–160 cm soil water | 13.9 | 0.4–45.1 |
|  |  |  |  | 160–200 cm soil water | 12.7 | 0.4–42.6 |
|  |  |  |  | Groundwater | 19.6 | 0.7–57.4 |
|  |  |  |  | River water | 16.9 | 0.6–52.4 |
|  |  | Plot 2 | Young | 0–40 cm soil water | 19.0 | 0.9–49.2 |
|  |  |  |  | 40–80 cm soil water | 14.8 | 0.4–47.9 |
|  |  |  |  | 80–120 cm soil water | 15.4 | 0.5–48.4 |
|  |  |  |  | 120–160 cm soil water | 15.5 | 0.5–47.5 |
|  |  |  |  | 160–200 cm soil water | 15.4 | 0.5–48.9 |
|  |  |  |  | Groundwater | 19.9 | 0.6–59.8 |
|  |  |  | Middle-aged | 0–40 cm soil water | 6.8 | 0.1–27.3 |
|  |  |  |  | 40–80 cm soil water | 18.0 | 0.6–56.6 |
|  |  |  |  | 80–120 cm soil water | 17.5 | 0.5–54.9 |
|  |  |  |  | 120–160 cm soil water | 18.1 | 0.6–57.1 |
|  |  |  |  | 160–200 cm soil water | 18.0 | 0.6–56.3 |
|  |  |  |  | Groundwater | 21.4 | 0.7–60.1 |
|  |  |  | Mature | 0–40 cm soil water | 14.8 | 0.4–46.5 |
|  |  |  |  | 40–80 cm soil water | 15.9 | 0.6–50.0 |
|  |  |  |  | 80–120 cm soil water | 15.9 | 0.6–49.4 |
|  |  |  |  | 120–160 cm soil water | 15.4 | 0.5–48.5 |
|  |  |  |  | 160–200 cm soil water | 15.0 | 0.5–46.9 |
|  |  |  |  | Groundwater | 23.1 | 0.8–66.9 |
|  |  | Plot 3 | Middle-aged | 0–40 cm soil water | 7.6 | 0.2–29.5 |
|  |  |  |  | 40–80 cm soil water | 17.3 | 0.6–54.6 |
|  |  |  |  | 80–120 cm soil water | 17.7 | 0.5–54.4 |
|  |  |  |  | 120–160 cm soil water | 17.8 | 0.6–55.6 |
|  |  |  |  | 160–200 cm soil water | 18.2 | 0.6–56.1 |
|  |  |  |  | Groundwater | 21.6 | 0.7–63.7 |
|  |  |  | Mature | 0–40 cm soil water | 6.6 | 0.1–26.1 |
|  |  |  |  | 40–80 cm soil water | 17.3 | 0.5–54.8 |
|  |  |  |  | 80–120 cm soil water | 17.8 | 0.6–54.9 |
|  |  |  |  | 120–160 cm soil water | 18.0 | 0.6–54.9 |
|  |  |  |  | 160–200 cm soil water | 18.3 | 0.6–55.7 |
|  |  |  |  | Groundwater | 21.9 | 0.7–62.6 |
|  | F | Plot 1 | Middle-aged | 0–40 cm soil water | 11.2 | 0.6–54.1 |
|  |  |  |  | 40–80 cm soil water | 12.8 | 0.1–33.8 |
|  |  |  |  | 80–120 cm soil water | 13.3 | 0.6–54.9 |
|  |  |  |  | 120–160 cm soil water | 14.1 | 0.1–34.3 |
|  |  |  |  | 160–200 cm soil water | 15.4 | 0.1–35.1 |
|  |  |  |  | Groundwater | 16.7 | 0.5–58.3 |
|  |  |  |  | River water | 16.4 | 0.5–57.3 |
|  |  |  | Mature | 0–40 cm soil water | 11.8 | 0.3–40.1 |
|  |  |  |  | 40–80 cm soil water | 12.3 | 0.4–42.6 |
|  |  |  |  | 80–120 cm soil water | 12.7 | 0.4–43.3 |
|  |  |  |  | 120–160 cm soil water | 13.4 | 0.4–43.9 |
|  |  |  |  | 160–200 cm soil water | 14.4 | 0.4–47.2 |
|  |  |  |  | Groundwater | 17.8 | 0.6–55.7 |
|  |  |  |  | River water | 17.6 | 0.6–55.7 |
|  |  | Plot 3 | Young | 0–40 cm soil water | 14.0 | 0.5–44.9 |
|  |  |  |  | 40–80 cm soil water | 12.8 | 0.4–41.3 |
|  |  |  |  | 80–120 cm soil water | 14.4 | 0.5–45.8 |
|  |  |  |  | 120–160 cm soil water | 14.2 | 0.5–45.1 |
|  |  |  |  | 160–200 cm soil water | 13.7 | 0.4–45.0 |
|  |  |  |  | Groundwater | 16.0 | 0.5–49.4 |
|  |  |  |  | River water | 14.9 | 0.4–47.4 |
|  |  |  | Middle-aged | 0–40 cm soil water | 16.4 | 0.6–51.6 |
|  |  |  |  | 40–80 cm soil water | 12.2 | 0.3–42.0 |
|  |  |  |  | 80–120 cm soil water | 13.9 | 0.4–46.1 |
|  |  |  |  | 120–160 cm soil water | 12.3 | 0.3–41.7 |
|  |  |  |  | 160–200 cm soil water | 11.5 | 0.3–40.0 |
|  |  |  |  | Groundwater | 16.7 | 0.5–53.0 |
|  |  |  |  | River water | 17.0 | 0.5–53.7 |
|  |  |  | Mature | 0–40 cm soil water | 11.8 | 0.3–40.2 |
|  |  |  |  | 40–80 cm soil water | 12.9 | 0.4–42.1 |
|  |  |  |  | 80–120 cm soil water | 13.6 | 0.4–45.0 |
|  |  |  |  | 120–160 cm soil water | 13.5 | 0.4–44.7 |
|  |  |  |  | 160–200 cm soil water | 13.9 | 0.5–45.0 |
|  |  |  |  | Groundwater | 18.4 | 0.6–57.0 |
|  |  |  |  | River water | 15.8 | 0.5–50.1 |
| July 2024 (non-EWC) | DW | Plot 1 | Young | 0–40 cm soil water | 15.9 | 0.8–54.7 |
|  |  |  |  | 40–80 cm soil water | 16.1 | 0.4–49.7 |
|  |  |  |  | 80–120 cm soil water | 17.9 | 0.5–52.2 |
|  |  |  |  | 120–160 cm soil water | 17.4 | 0.5–50.0 |
|  |  |  |  | 160–200 cm soil water | 16.8 | 0.6–49.4 |
|  |  |  |  | Groundwater | 15.9 | 0.6–49.5 |
|  |  |  | Middle-aged | 0–40 cm soil water | 12.9 | 0.4–43.4 |
|  |  |  |  | 40–80 cm soil water | 15.8 | 0.5–49.4 |
|  |  |  |  | 80–120 cm soil water | 18.0 | 0.7–55.4 |
|  |  |  |  | 120–160 cm soil water | 16.9 | 0.6–52.6 |
|  |  |  |  | 160–200 cm soil water | 16.5 | 0.5–52.1 |
|  |  |  |  | Groundwater | 19.9 | 0.8–53.7 |
|  |  |  | Mature | 0–40 cm soil water | 14.8 | 0.5–49.2 |
|  |  |  |  | 40–80 cm soil water | 14.8 | 0.6–49.6 |
|  |  |  |  | 80–120 cm soil water | 16.5 | 0.6–54.2 |
|  |  |  |  | 120–160 cm soil water | 16.7 | 0.6–53.8 |
|  |  |  |  | 160–200 cm soil water | 17.6 | 0.6–51.1 |
|  |  |  |  | Groundwater | 19.7 | 0.5–49.1 |
|  |  | Plot 2 | Young | 0–40 cm soil water | 15.8 | 0.6–49.9 |
|  |  |  |  | 40–80 cm soil water | 16.6 | 0.5–52.3 |
|  |  |  |  | 80–120 cm soil water | 16.8 | 0.5–51.8 |
|  |  |  |  | 120–160 cm soil water | 16.5 | 0.6–50.3 |
|  |  |  |  | 160–200 cm soil water | 17.0 | 0.5–51.9 |
|  |  |  |  | Groundwater | 17.4 | 0.6–53.1 |
|  |  |  | Middle-aged | 0–40 cm soil water | 10.9 | 0.3–36.6 |
|  |  |  |  | 40–80 cm soil water | 17.1 | 0.5–43.0 |
|  |  |  |  | 80–120 cm soil water | 17.6 | 0.7–62.6 |
|  |  |  |  | 120–160 cm soil water | 17.7 | 0.7–63.3 |
|  |  |  |  | 160–200 cm soil water | 19.1 | 0.7–62.7 |
|  |  |  |  | Groundwater | 17.6 | 0.8–65.4 |
|  |  |  | Mature | 0–40 cm soil water | 13.2 | 0.4–43.2 |
|  |  |  |  | 40–80 cm soil water | 17.3 | 0.5–52.1 |
|  |  |  |  | 80–120 cm soil water | 17.4 | 0.6–54.9 |
|  |  |  |  | 120–160 cm soil water | 17.3 | 0.5–52.9 |
|  |  |  |  | 160–200 cm soil water | 17.5 | 0.5–54.0 |
|  |  |  |  | Groundwater | 17.3 | 0.6–50.9 |
|  |  | Plot 3 | Middle-aged | 0–40 cm soil water | 14.3 | 0.5–53.5 |
|  |  |  |  | 40–80 cm soil water | 14.1 | 0.5–51.8 |
|  |  |  |  | 80–120 cm soil water | 16.1 | 0.6–54.1 |
|  |  |  |  | 120–160 cm soil water | 17.4 | 0.8–60.4 |
|  |  |  |  | 160–200 cm soil water | 20.3 | 0.8–61.6 |
|  |  |  |  | Groundwater | 17.8 | 1.0–70.6 |
|  |  |  | Mature | 0–40 cm soil water | 15.1 | 0.6–57.2 |
|  |  |  |  | 40–80 cm soil water | 16.1 | 0.6–57.2 |
|  |  |  |  | 80–120 cm soil water | 15.8 | 0.5–60.3 |
|  |  |  |  | 120–160 cm soil water | 16.0 | 0.6–59.9 |
|  |  |  |  | 160–200 cm soil water | 19.1 | 0.7–57.5 |
|  |  |  |  | Groundwater | 18.0 | 0.8–69.6 |
|  | F | Plot 1 | Middle-aged | 0–40 cm soil water | 19.4 | 0.7–55.6 |
|  |  |  |  | 40–80 cm soil water | 16.3 | 0.5–51.5 |
|  |  |  |  | 80–120 cm soil water | 14.8 | 0.4–47.6 |
|  |  |  |  | 120–160 cm soil water | 16.1 | 0.6–50.8 |
|  |  |  |  | 160–200 cm soil water | 16.3 | 0.6–51.7 |
|  |  |  |  | Groundwater | 17.2 | 0.6–53.9 |
|  |  |  | Mature | 0–40 cm soil water | 12.6 | 0.4–40.9 |
|  |  |  |  | 40–80 cm soil water | 13.5 | 0.4–43.6 |
|  |  |  |  | 80–120 cm soil water | 17.3 | 0.5–53.9 |
|  |  |  |  | 120–160 cm soil water | 19.1 | 0.6–56.0 |
|  |  |  |  | 160–200 cm soil water | 19.4 | 0.6–53.7 |
|  |  |  |  | Groundwater | 18.1 | 0.6–56.8 |
|  |  | Plot 3 | Young | 0–40 cm soil water | 16.0 | 0.5–51.0 |
|  |  |  |  | 40–80 cm soil water | 16.0 | 0.5–50.2 |
|  |  |  |  | 80–120 cm soil water | 16.3 | 0.5–50.1 |
|  |  |  |  | 120–160 cm soil water | 16.6 | 0.6–50.8 |
|  |  |  |  | 160–200 cm soil water | 16.8 | 0.5–51.8 |
|  |  |  |  | Groundwater | 18.4 | 0.7–54.8 |
|  |  |  | Middle-aged | 0–40 cm soil water | 16.3 | 0.5–54.2 |
|  |  |  |  | 40–80 cm soil water | 15.7 | 0.5–49.5 |
|  |  |  |  | 80–120 cm soil water | 16.2 | 0.6–51.3 |
|  |  |  |  | 120–160 cm soil water | 16.6 | 0.5–52.8 |
|  |  |  |  | 160–200 cm soil water | 16.7 | 0.5–53.1 |
|  |  |  |  | Groundwater | 18.4 | 0.7–56.1 |
|  |  |  | Mature | 0–40 cm soil water | 14.6 | 0.6–53.4 |
|  |  |  |  | 40–80 cm soil water | 16.7 | 0.6–53.4 |
|  |  |  |  | 80–120 cm soil water | 16.6 | 0.7–58.9 |
|  |  |  |  | 120–160 cm soil water | 16.9 | 0.7–57.4 |
|  |  |  |  | 160–200 cm soil water | 19.6 | 0.7–59.4 |
|  |  |  |  | Groundwater | 15.5 | 0.9–65.8 |
